# The ketogenic diet alters microbiome‐metabolome profiles to improve West syndrome therapy

**DOI:** 10.1002/ped4.70027

**Published:** 2025-11-19

**Authors:** Gan Xie, Qian Zhou, Jianxiang Liao, Yuejie Zheng, Wenjian Wang, Kunling Shen

**Affiliations:** ^1^ Department of Respiratory Medicine Beijing Children's Hospital Capital Medical University National Center for Children's Health Beijing China; ^2^ Department of Computer Science City University of Hong Kong Hong Kong China; ^3^ Department of Neurology Shenzhen Children's Hospital Shenzhen China; ^4^ Department of Respiratory Medicine Shenzhen Children's Hospital Shenzhen China

**Keywords:** Epilepsy, Gastrointestinal microbiota, Ketogenic diet, Metabolomics

## Abstract

**Importance:**

The ketogenic diet (KD) is effective in managing epilepsy, particularly West syndrome (WS); however, the role of gut microbiome (GM) and metabolome in its efficacy remains unclear. Understanding these mechanisms could optimize the KD for WS treatment.

**Objective:**

To identify microbiome‐metabolome signatures associated with KD efficacy in WS by analyzing changes in GM composition and metabolic pathways.

**Methods:**

Fecal samples were collected from WS patients (*n* = 16) and healthy children (*n* = 24). Metagenome and metabolome analyses were performed to assess GM composition and metabolic profiles.

**Results:**

WS patients showed GM imbalances compared to healthy children. Disease status contributed sufficiently to the GM. The abundance of *Bacteroides*, *Parabacteroides*, and *Faecalibacterium* was lower in WS (3.30% vs. 39.86%, *P*‐adj = 0.140; 0.14% vs. 0.73%, *P*‐adj = 0.023; 0.04% vs. 1.35%, *P*‐adj = 0.018), whereas *Bifidobacterium* and *Escherichia* were higher (6.08% vs. 2.23%, *P*‐adj = 0.140; 7.57% vs. 0.15%, *P*‐adj < 0.001). After KD, *Parabacteroides* (particularly *P. distasonis*) and *Bacteroides* (particularly *B. fragilis*) increased (0.14% vs. 0.35%, *P*‐adj = 0.034; 3.30% vs. 21.18%, *P*‐adj = 0.380); *Bifidobacterium* (particularly *B. breve*) and *Escherichia* (particularly *E. coli*) decreased from 6.08% and 7.57% to 1.24% and 2.52%, respectively. Kyoto Encyclopedia of Genes and Genomes pathway analysis demonstrated that ATP‐binding cassette (ABC) transporters, fatty acid biosynthesis, tyrosine metabolism, and other pathways were significantly altered in patients with WS, and these alterations were reversed following ketogenic diet (KD) consumption. The KD also altered intestinal metabolites. Integrative analysis of microbial features, gene functions, and metabolites revealed that *Bacteroides* species and *P. distasonis* were significantly associated with ABC transporters, alanine aspartate and glutamate metabolism, and negatively correlated with 3‐sulfinoalanine, suggesting potential regulatory roles in metabolic pathways.

**Interpretation:**

KD induces significant shifts in GM composition and metabolic pathways, which may contribute to its therapeutic efficacy in WS. The restoration of *Bacteroides* and *Parabacteroides* dominance, alongside alterations in gene functions and neurotransmitter‐related metabolites, suggests a potential mechanism for the antiepileptic effects of KD.

## Graphical abstract

The ketogenic diet alleviates West syndrome by reshaping gut microbiota (increasing *Bacteroides*, *Parabacteroides*, and reducing *Escherichia* and *Bifidobacterium*), and modulating metabolites—including anticonvulsant lipids (e.g., capric acid), suppressed harmful lipids (e.g., 2‐methylbutyroylcarnitine), and amino acids and analogs (e.g., 3‐sulfinoalanine)—linked to energy metabolism and neurotransmitter regulation, highlighting microbiome‐metabolome crosstalk in antiepileptic efficacy.

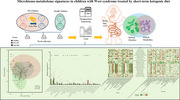

## INTRODUCTION

Epilepsy, a prevalent neurological disorder, frequently emerges during childhood.^1^ Among infants with refractory epilepsy, a notable subset is diagnosed with West syndrome (WS).^2^ Patients with WS typically exhibit three main clinical manifestations: convulsive seizures, irregular electroencephalographic patterns, and psychomotor regression or retardation.^3^ These symptoms can lead to developmental delays or mortality, affecting up to 30% of severe cases.^4^ Although antiepileptic drugs (AEDs) are the preferred treatments for epilepsy, their efficacy in patients with WS is often limited. Even when combined with adrenocorticotrophic hormone (ACTH) or vigabatrin, 33%–56% of patients continue to experience seizures.^5^

Several clinical studies demonstrated the effectiveness of the ketogenic diet (KD) in treating WS patients.^6^ A prospective study reported that more than 50% of children with WS experienced a reduction in seizure frequency within 2 weeks of KD consumption.^7^ In particular, the median duration between the start of KD consumption and a reduction in epileptic seizures is approximately 5 days.^8^ A systematic review of 341 patients with WS consuming the KD found that the seizure frequency declined by at least 50% in 64.7% of patients, with 34.61% achieving relief from spasticity.^9^ Guidelines suggest that KD feeding can reduce seizures in more than 70% of patients with WS, especially in children resistant to ACTH and aminohexanoic acid.^10^ However, the antiepileptic mechanism of the KD remains incompletely understood.^11^

It is well established that the diet can alter the composition of the gut microbiota (GM) within 1 day.^12^ The KD, characterized by high fat and low carbohydrate content, can significantly and rapidly reshape the GM. Previous studies utilizing 16S rRNA sequencing revealed disease‐specific GM patterns in patients with refractory epilepsy.^13^, ^14^ Furthermore, the antiepileptic properties of certain probiotics have been identified.^15^ Metabolomic studies have consistently identified strong associations between microbe‐related metabolites and various neurological disorders, such as depression, Parkinson's disease, Alzheimer's disease, and ischemic stroke.^16^, ^17^, ^18^, ^19^

In this study, we investigated the GM patterns in patients with WS using metagenomics and metabolomics to clarify the influence of the KD on gut microbial ecology and identify potential factors contributing to epileptic control.

## METHODS

### Ethical approval

The study was approved by the Ethics Committee of Shenzhen Children's Hospital (registration number 2017(005)). Written informed consent was obtained from the guardians of all participating children.

### Subject enrollment

We enrolled patients diagnosed with WS at the Department of Pediatric Neurology of Shenzhen Children's Hospital (Shenzhen, Guangdong Province, China) between January 2015 and April 2020. The detailed enrollment criteria were as follows: 1) meeting the WS definition according to the International League Against Epilepsy Commission^20^; 2) patients were administered KD following informed consent, after experiencing inadequate responses to conventional AEDs; 3) exclusion of patients who had been exposed to antibiotics or probiotics within the 4 weeks prior to sampling; and 4) exclusion of patients with contraindications to the KD. Age‐matched healthy control subjects were selected from a previous study. Healthy controls were defined as individuals with no known history of epilepsy, no chronic medical conditions (e.g., metabolic or neurological disorders), and no prior use of psychoactive medications. All control participants were reported to be in good general health and maintained a regular, balanced diet.^21^

### KD treatment and fecal sample collection

The classic KD protocol was implemented. During the first week, all patients received Qitong Milk (Shenzhen Jielikang Biotechnology Co., Ltd.) as the sole KD formula, with a fat‐to‐(protein+carbohydrate) ratio ranging from 2:1 to 4:1, individually adjusted based on patient tolerance. The formula primarily contains vegetable oil, milk protein, and soy protein. After discharge, caregivers prepared supplementary meals at home under the supervision of certified ketogenic dietitians. Patients recorded their daily blood glucose and ketone levels and seizure frequencies during KD consumption in the hospital. Fecal samples were collected from the patients before (Pre_KD group) and after 1 week of KD consumption (Post_KD group) and promptly stored at −80°C within 1 hour after collection. Seizure frequency was assessed based on parental seizure diaries. The average daily seizure count during the 28 days prior to KD initiation was used as a baseline and was compared with the frequency recorded 1 week after diet onset.

### DNA extraction and sequencing

Microbial DNA was extracted using the QIAamp DNA Stool Kit (Qiagen, Hilden, Germany) according to the manufacturer's protocol. The DNA quality was measured via a NanoDrop (Thermo Fisher Scientific, Waltham, MA, USA), and the quantity was assessed by Qubit (Thermo Fisher Scientific). Eligible DNA fragments were selected through agarose gel electrophoresis. The metagenomic library was generated using a NEBNext Ultra DNA Library Prep Kit (New England Biolabs, Ipswich, MA, USA), and high‐throughput sequencing with 2 × 150‐bp paired‐end reads was performed using the Illumina NovaSeq platform (Illumina, San Diego, CA, USA).

### Metagenome analysis

Raw sequencing reads were quality‐filtered using SOAPnuke (v1.4.2) to remove low‐quality sequences, adapter contaminants, and primer sequences. The cleaned reads were then aligned to the human reference genome (GRCh38.p13) using Bowtie2 (v2.3.5.1) to exclude host‐derived DNA.

Taxonomic profiling was performed using Kraken2 (v2.8.0),^22^ with microbial abundance estimation refined by Bracken to improve resolution at lower taxonomic levels. Microbial relative abundances were calculated based on the number of reads assigned to each taxon, resulting in a high‐resolution taxonomic profile across all samples.

For functional annotation, filtered reads were mapped to the gut microbiome catalog^23^—a comprehensive reference gene set derived from cultured and uncultured gut microbes—using bowtie2. Gene‐level abundance was quantified by counting the number of reads uniquely aligned to each annotated gene. These counts were normalized to counts per million (CPM) to account for differences in sequencing depth across samples. Functional profiles were inferred by mapping individual genes to Kyoto Encyclopedia of Genes and Genomes (KEGG) Orthology (KO) entries based on pre‐existing annotations provided by the catalog. Higher‐level functional modules and pathways were reconstructed by aggregating CPM‐normalized gene abundances within each KEGG pathway.

### Untargeted metabolic profiling

Samples (25 mg) were extracted with 500 µL of a solution (acetonitrile:methanol:water = 2:2:1) containing isotopically labeled internal standards. The mixture was vortexed (30 s, 35 Hz), homogenized (4 min), and sonicated (5 min, ice‐water bath). The supernatant, along with quality‐control samples, was analyzed using ultrahigh‐performance liquid chromatography (1290 Infinity series, Agilent Technologies) coupled with a QE mass spectrometer. MS/MS spectra were acquired using Xcalibur software (v4.0.27, Thermo Fisher Scientific). Raw data were converted to mzXML format (ProteoWizard) and processed for peak detection, alignment, and integration using an in‐house program. Metabolites were annotated using an in‐house MS2 database (BiotreeDB, cutoff = 0.3) and concentrations were determined by peak area normalization with internal standards.

### Statistical analysis

Genus‐ and species‐level taxa with a median relative abundance of ≥ 0.05% across samples were retained for downstream analysis. The top 10 genera, and top 20 species and gene functions (ranked by median abundance within each group) were defined as dominant for visualization and statistical testing. Principal component analysis (PCA) was performed on Hellinger‐transformed species‐level relative abundance data to explore overall compositional patterns and was visualized using the ade4 package. Permutational multivariate analysis of variance (PERMANOVA, via adonis2 in the vegan package, 9999 permutations) was applied to assess the association between clinical phenotypes and baseline gut microbiota structure. Alpha diversity indices (Shannon, Richness, and Pielou's evenness) were compared across groups using the Kruskal‐Wallis test. Pairwise comparisons were conducted using the unpaired Wilcoxon rank‐sum test for Healthy versus Pre_KD (independent groups) and paired Wilcoxon signed‐rank test for Pre_KD vs. Post_KD (within‐subject changes). Beta diversity was analyzed using principal coordinate analysis (PCoA) based on Bray‐Curtis dissimilarity and Aitchison distance (vegdist in vegan), and the group separation was statistically evaluated using PERMANOVA (adonis2, 9999 permutations). PERMANOVA *R* ^2^ and *P*‐values are reported directly on ordination plots. All *P*‐values from multiple comparisons were adjusted using the false discovery rate (FDR) method; adjusted *P* (*P*‐adj) < 0.05 was considered statistically significant. Orthogonal projections to latent structures discriminant analysis (OPLS‐DA) was performed to identify discriminant metabolites between groups. Metabolites with variable importance in projection (VIP) > 1 and FDR‐adjusted *P* < 0.05 (Wilcoxon signed‐rank test) were selected as significantly differential. Comparisons of clinical phenotypes between groups were performed using Student's *t*‐test (for normally distributed continuous variables) or Fisher's exact test (for categorical variables). Spearman's rank correlation (via Hmisc package) was used to assess associations among microbial taxa, functional pathways, and metabolites.

## RESULTS

### Clinical characteristics and data summary

The study originally aimed to enroll 30 patients and 30 controls. However, due to challenges in sample collection—particularly constipation in pediatric patients—a total of 16 children with WS were ultimately included. Among them, stool samples were collected both before and 1 week after KD initiation from 12 patients. Two patients contributed only the Pre_KD sample, and two others contributed only the Post_KD sample. Of the initially targeted population of 30 healthy controls, 24 were included to match the age distribution of children with WS (Table 1). There were no statistically significant differences in demographic or anthropometric characteristics, including age, sex, height, and weight, between the patient group and healthy controls (Table 1 and Table S1). The AEDs remained unchanged during the first week of KD consumption. Following 1 week of KD feeding, 10 patients experienced a greater than 50% reduction in seizure frequency according to the epilepsy diary recorded by the patient's parents, with two becoming seizure‐free, whereas the seizure frequency was reduced by less than 50% in the remaining six patients (Table S1). Upon metagenomic taxonomic classification, the microbial taxonomy profiles comprised 1421 genera and 4992 species. However, a large proportion of the sequence data (43.26% ± 17.64%) remained unclassified from known microbial sequences. Bacterial DNA predominated (>99.5%) compared to viral or fungal DNA. In total, 3012 metabolites were identified by untargeted metabolite assays.

**TABLE 1:** Characteristics of patients with West syndrome (WS) and healthy children

Characteristics	Patients with WS (*n* = 16)	Healthy children (*n* = 24)	*P*‐value
Sex			
Male	5	9	0.746
Female	11	15	
Age (years)	1.5 ± 0.8	1.8 ± 0.6	0.120
Weight (kg)	10.5 ± 5.1	12.2 ± 3.7	0.276
Height (cm)	79.9 ± 7.7	84.4 ± 9.0	0.100

### The GM structure differed between children with WS and healthy children

**FIGURE 1:**
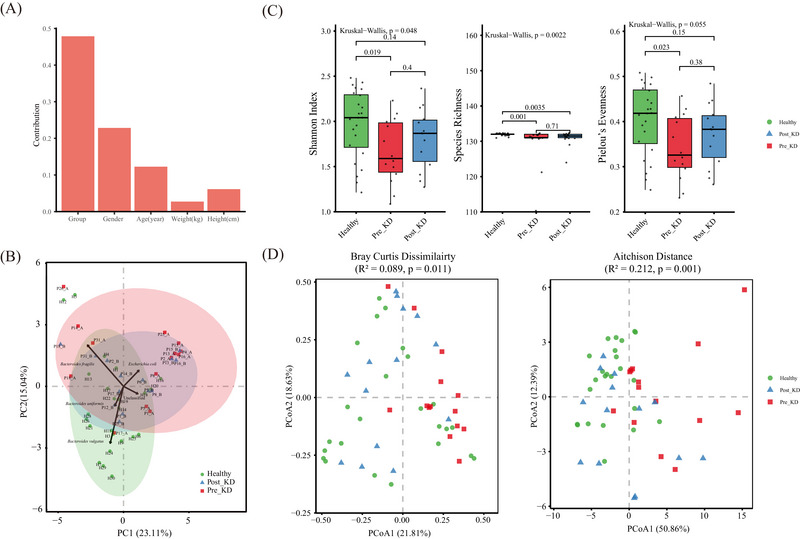
Gut microbiota structure in patients with West syndrome (WS) before and after ketogenic diet (KD) therapy, compared to healthy children. (A) PERMANOVA was used to assess the contribution of clinical factors (e.g., group, gender, and age) to gut microbiota variation; bar height represents the sum of squared distances (SSD). (B) Principal component analysis (PCA) was performed on Hellinger‐transformed species‐level relative abundances. Each point represents a sample, colored by group (green: Healthy; blue: Post_KD; red: Pre_KD). Light‐colored ellipses indicate 90% confidence regions. Black arrows represent the top five most influential taxa (based on correlation with PC1/PC2); arrow direction and length reflect their contribution to ordination space. PC1 and PC2 explain 23.11% and 15.04% of total variance, respectively. (C) Alpha diversity metrics (Shannon index, species richness, Pielou's evenness) are presented as boxplots showing median, interquartile range (IQR), and whiskers extending to 1.5×IQR. Individual sample values are overlaid as jittered dots. Groups were compared using the Kruskal‐Wallis test, followed by pairwise Wilcoxon rank‐sum tests with false discovery rate (FDR) adjustment; *P* < 0.05 was considered statistically significant. (D) Principal coordinate analysis (PCoA) based on Bray‐Curtis dissimilarity (left) and Aitchison distance (right). Group separation was assessed using PERMANOVA (adonis2, 9999 permutations); *R* ^2^ and *P*‐values are reported in the inset. Each symbol represents an individual sample, colored by group (green: Healthy; blue: Post_KD; red: Pre_KD). Pre_KD: baseline samples collected before KD initiation; Post_KD: samples collected after 1 week of KD therapy.

The results of PERMANOVA indicated that the disease status sufficiently contributed to the GM (sum of squared differences = 0.479, *R* ^2^ = 0.117, *P* = 0.008), whereas other clinical indicators or phenotypes were not significantly associated with the GM (Figure 1A). PCA and PCoA showed distinct clustering of the GM composition: the Pre_KD group exhibited greater dispersion, whereas healthy controls were tightly clustered (Figure 1B, D – left panel) (Figure 1B). This pattern was consistent with lower microbial alpha‐diversity in the Pre_KD group, as evidenced by reduced Shannon index (1.59 vs. 2.04, *P* = 0.019) (Figure 1C). The other two alpha diversity indices (species richness and Pielou's evenness) were also lower in the Pre_KD group, indicating that the GM of healthy individuals was more abundant and more evenly distributed, and that these indices were increased by KD consumption (Figure 1C).

### Imbalanced microbial profiling and functional pathway in children with WS and healthy children

At the phylum level, the dominant bacteria were *Bacteroidetes* (Pre_KD vs. Healthy: 4.50% vs. 49.11%), followed by *Firmicutes* (9.45% vs. 9.23%), and *Actinobacteria* (6.20% vs. 2.94%), and their abundance was not significantly different between these groups (Table S2 and Figure S1). However, *Proteobacteria* were significantly enriched in patients (17.59% vs. 1.17%, *P*‐adj < 0.001, Table S2 and Figure S1).

**FIGURE 2:**
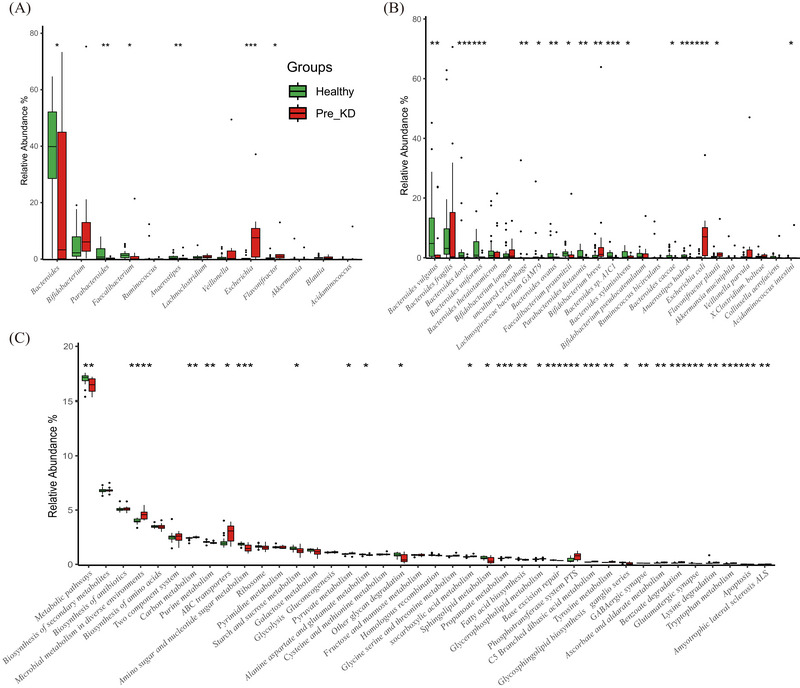
Comparative analysis of gut microbiota composition and functional profiles between the Pre_KD and healthy control groups. (A) Relative abundance of dominant bacterial genera (median ≥0.05% across samples), (B) dominant species, and (C) KEGG pathways (top 10, 20, and 20, respectively by mean abundance per group). Data are shown as boxplots, with median (central line), interquartile range (box), and outliers (points). Statistical comparisons were performed using the unpaired Wilcoxon rank‐sum test to assess differences between independent groups (Healthy vs. Pre_KD). Significance was adjusted for multiple testing using the false discovery rate (FDR); *P < 0.05, **P < 0.01, ***P < 0.001 (FDR‐adjusted). Pre_KD, samples collected from patients before initiation of ketogenic diet therapy.

The top five genera in patients were *Escherichia* (Pre_KD vs. Healthy: 7.57% vs. 0.15%, *P*‐adj < 0.001), *Bifidobacterium* (6.08% vs. 2.23%, *P*‐adj = 0.140), *Bacteroides* (3.30% vs. 39.86%, *P*‐adj = 0.140), *Flavonifractor* (1.01% vs. 0.30%, *P*‐adj = 0.068), and *Lachnoclostridium* (0.82% vs. 0.50%, *P*‐adj = 0.670, Figure 2A and Table S3). The other three genera, namely *Parabacteroides* (0.14% vs. 0.73%, *P*‐adj = 0.023), *Anaerostips* (0.07% vs. 0.40%, *P*‐adj = 0.028), and *Faecalibacterium* (0.04% vs. 1.35%, *P*‐adj = 0.018) were more abundant in healthy controls (Figure 2A, Table S3).

Species‐level analysis revealed that *Bacteroides vulgatus* (Pre_KD vs. Healthy: 0.03% vs. 4.78%, *P*‐adj = 0.005), *Bacteroides uniformis* (0.10% vs. 0.85%, *P*‐adj = 0.001), *Bacteroides dorei* (0.02% vs. 0.67%, *P*‐adj < 0.001), *Bacteroides sp*. *A1C1* (0.03% vs. 0.53%, *P*‐adj < 0.001), *Bacteroides ovatus* (0.03% vs. 0.20%, *P*‐adj = 0.023), *Faecalibacterium prausnitzii* (0.04% vs. 1.35%, *P*‐adj = 0.018), *Parabacteroides distasonis* (0.08% vs. 0.42%, *P*‐adj = 0.021), and *Anaerostipes hadrus* (0.02% vs. 0.38%, *P*‐adj = 0.002) were significantly more abundant in healthy controls than in patients with WS (Figure 2B and Table S4). *Bacteroides fragilis* (0.31% vs. 3.11%, *P*‐adj = 0.150) was more abundant in healthy controls than in WS patients, albeit without significance (Figure 2B and Table S4). Among *Bifidobacterium* species, *Bifidobacterium breve* (1.12% vs. 0.17%, *P*‐adj = 0.024) was more abundant in patients. Other species with significant differences in abundance between healthy controls and patients with WS were *Escherichia coli* (7.00% vs. 0.14%, *P*‐adj < 0.001, Figure 2B and Table S4).

KEGG pathway analysis demonstrated that the gene abundance of 84 metabolic pathways was significantly changed in patients with WS. Pathways with decreased abundance in patients included GABAergic synapse, starch and sucrose metabolism, alanine aspartate and glutamate metabolism, sphingolipid metabolism, fatty acid biosynthesis, base excision repair, glycosphingolipid biosynthesis ganglio series, glutamatergic synapse, apoptosis (Figure 2C and Table S5); meanwhile, carbon metabolism, ATP‐binding cassette (ABC) transporters, pyruvate metabolism, oxocarboxylic acid metabolism, propanoate metabolism, glycerophospholipid metabolism, phosphotransferase system, tyrosine metabolism, benzoate degradation, lysine degradation, tryptophan metabolism, and amyotrophic lateral sclerosis were enriched in the GM of patients with WS (Figure 2C and Table S5).

### KD consumption restored the imbalanced gut micro‐ecology

After 1 week of KD intervention, the GM structure shifted toward that of healthy individuals. PCA and PCoA plots indicated that Post_KD samples became more clustered, resembling the healthy control group (Figure 1B and 1D). Alpha‐diversity indices increased after KD, with the Shannon index reaching levels comparable to those of healthy controls (Post_KD: 1.87 vs. Healthy: 2.04, *P*‐adj = 0.140; Figure 1C). Moreover, PERMANOVA confirmed a significant reduction in GM dissimilarity following KD (*R* ^2^ = 0.089, *P* = 0.011) (Figure 1D, left panel), indicating a restoration of microbial community stability. Aitchison distance‐based PCoA further supported these findings, showing improved clustering and reduced inter‐sample variability Post_KD (*R* ^2^ = 0.212, *P* = 0.001) (Figure 1D, right panel).

**FIGURE 3:**
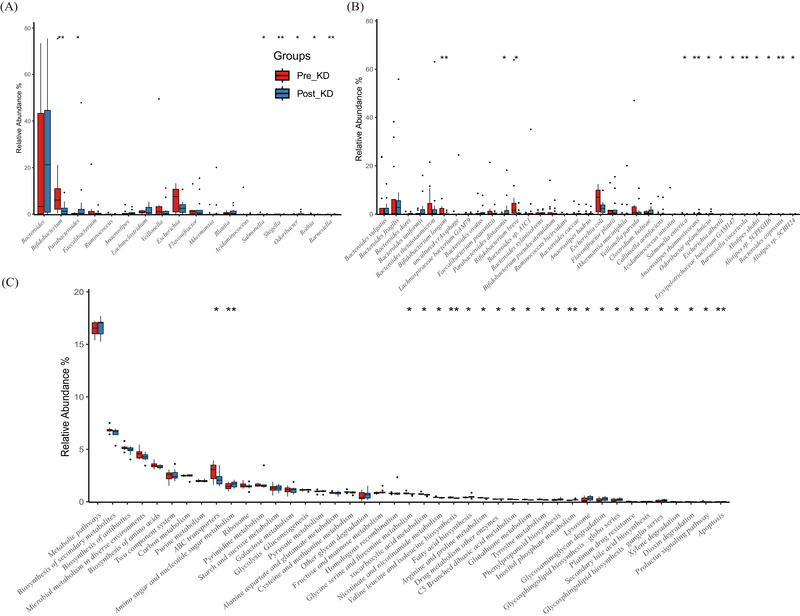
Paired comparative analysis of gut microbiota composition and functional profiles between Pre_KD and Post_KD groups. (A) Relative abundance of dominant bacterial genera, (B) dominant species, and (C) KEGG pathways (top 10, 20, and 20, respectively, by mean abundance per group). Data are shown as boxplots, with median (central line), interquartile range (box), and outliers (points). Statistical comparisons were performed using the Wilcoxon signed‐rank test for within‐subject changes (Pre_KD vs. Post_KD). Significance was adjusted for multiple testing using the false discovery rate (FDR); *P < 0.05, **P < 0.01, ***P < 0.001 (FDR‐adjusted). Pre_KD, samples collected before ketogenic diet initiation; Post_KD, samples collected after 1 week of ketogenic diet therapy.

The pairwise statistical tests before and after ketogenesis showed that seven genera were significantly changed, especially *Bifidobacterium* (Pre_KD vs. Post_KD: 6.08% vs. 1.24%, *P*‐adj = 0.005) and *Parabacteroides* (0.14% vs. 0.35%, *P*‐adj = 0.034, Figure 3A and Table S6). The proportion of *Escherichia* decreased from 7.57% to 2.52% after KD, and that of *Bacteroides* increased from 3.30% to 21.18%, although the difference was not statistically significant. And their median levels were similar to those in the healthy control group (Figure 3A).

At the species level, the relative abundances of 13 bacterial species changed significantly after KD consumption, including sharp decreases in the abundance of *B. breve* (1.50% vs. 0.17%, *P*‐adj = 0.012), *Bifidobacterium longum* (0.18% vs. 0.07%, *P*‐adj = 0.003), *Salmonella enterica* (0.02% vs. 0.01%, *P*‐adj = 0.042) and an increase in the abundance of *P. distasonis* (0.08% vs. 0.19%, *P*‐adj = 0.027), *Anaerostipes rhamnosivorans* (0.02% vs. 0.03%, *P*‐adj = 0.009, Figure 3B and Table S7). The abundance of *B. fragilis* (8.79‐fold), *B. uniformis* (2.15‐fold), *Bacteroides xylanisolvens* (4.98‐fold), *B. vulgatus* (4.87‐fold), and *Akkermansia muciniphila* (1.75‐fold) was also dramatically changed by the KD intervention, but statistical significance was not achieved (Figure 3B, Table S7). After KD treatment, the abundance of *E. coli* decreased from 7.00% to 2.32% (*P*‐adj = 0.200).

The abundance of KEGG pathways, especially ABC transporters (*P*‐adj = 0.012), amino sugar and nucleotide sugar metabolism (*P*‐adj = 0.002), glycine serine and threonine metabolism (*P*‐adj = 0.016), oxocarboxylic acid metabolism (*P*‐adj = 0.016), nicotinate and nicotinamide metabolism (*P*‐adj = 0.042), valine leucine and isoleucine biosynthesis (*P*‐adj = 0.009), fatty acid biosynthesis (*P*‐adj = 0.021), arginine and proline metabolism (*P*‐adj = 0.042), C5‐branched dibasic acid metabolism (*P*‐adj = 0.034), glutathione metabolism (*P*‐adj = 0.021), tyrosine metabolism (*P*‐adj = 0.027), inositol phosphate metabolism (*P*‐adj = 0.007), lysosome (*P*‐adj = 0.016), glycosaminoglycan degradation (*P*‐adj = 0.034), glycosphingolipid biosynthesis globo series (*P*‐adj = 0.027), and glycosphingolipid biosynthesis ganglio series (*P*‐adj = 0.012) also recovered after the intervention (Figure 3C and Table S8).

**FIGURE 4:**
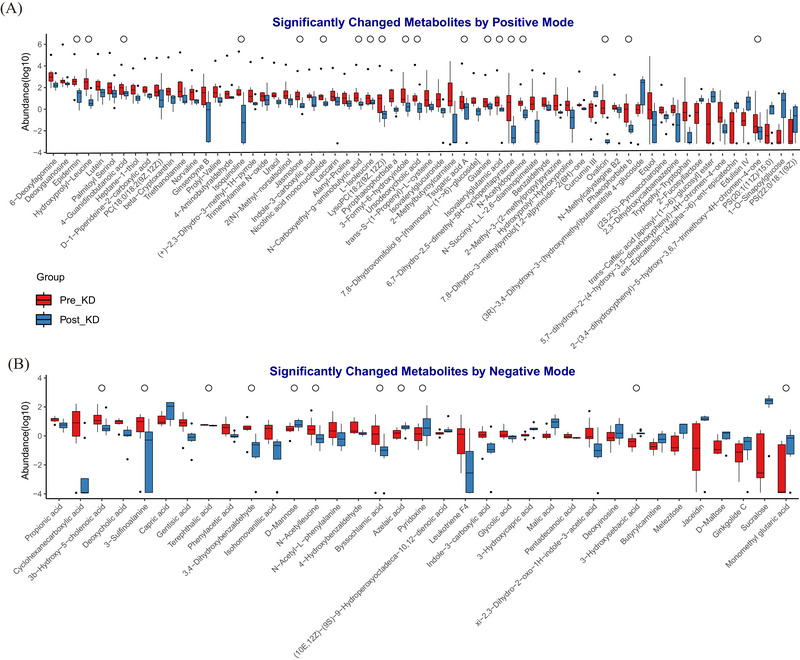
Paired comparative analysis of metabolite profiles between Pre_KD and Post_KD groups. (A) Significantly changed metabolites detected in positive ion mode; (B) Significantly changed metabolites detected in negative ion mode. Metabolite abundances were log‐transformed for improved visualization. Data are shown as boxplots, with median (central line), interquartile range (box), and outliers (points). “°” denotes metabolites with variable importance in projection (VIP) >1 in the OPLS‐DA model, indicating high discriminatory power between groups. Pre_KD, baseline samples collected before KD initiation; Post_KD, samples collected after 1 week of KD therapy.

The alteration of metabolites provided more direct evidence of the mechanism of KD. After combining OPLS‐DA and the Wilcoxon signed‐rank test with paired samples, a total of 30 metabolites were identified as significantly altered following KD consumption (VIP > 1 and *P*‐adj < 0.05). To expand the search for metabolites potentially associated with the KD and epilepsy, a less stringent criterion (*P*‐adj < 0.05, without VIP filtering) was applied. This approach identified 98 differentially abundant metabolites, among which 74 were enriched and 24 were suppressed in the Pre_KD group compared to those in the Post_KD group (Figure 4 and Table S9). These differential metabolites belong to 11 superclasses: lipid and lipid‐like molecules, organic acids and derivatives, organoheterocyclic compounds, organic oxygen compounds, organic nitrogen compounds, organohalogen compounds, organosulfur compounds, benzenoids, phenylpropanoids and polyketides, nucleosides, alkaloids and derivatives. In the lipid class, 2‐methylbutyroylcarnitine and deoxycholic acid, which are associated with epilepsy onset, were enriched in patients with WS before KD consumption. Of these, their abundance decreased sharply after the intervention (6.22 vs. <0.01, *P*‐adj = 0.039; 10.63 vs. 1.17, *P*‐adj = 0.008, Figure 4 and Table S9). Contrarily, eight lipid metabolites were enriched in the Post_KD group, and among them, capric acid and (3R)‐3,4‐dihydroxy‐3‐(hydroxymethyl)butanenitrile 4‐glucoside were linked to anticonvulsant effects. Among organic acids and derivatives, the concentrations of two medium‐chain fatty acids (3‐hydroxysebacic acid and 3‐hydroxycapric acid) and one beta‐hydroxy acid (malic acid) were increased by KD consumption; Among amino acids and analogs, including hydroxyprolyl‐leucine, 3‐sulfinoalanine, N‐acetyl‐L‐phenylalanine, N‐acetylleucine, norvaline, tryptophyl‐tryptophan, N‐succinyl‐L,L‐2,6‐diaminopimelate, 4‐guanidinobutanoic acid, N‐carboxyethyl‐γ‐aminobutyric acid, and 4‐hydroxybenzaldehyde, all of their levels tended to decrease after the KD intervention (Figure 4 and Table S9). Metabolites of benzenoids were decreased in the Post_KD group, including N‐acetyldopamine, benzaldehyde, isohomovanillic acid, 3,4‐dihydroxybenzaldehyde, gentisic acid, and phenylacetic acid, whereas the concentration of curcumin III was increased by KD consumption (Figure 4 and Table S9).

Eight metabolites were reported for phenylpropanoids and polyketides, including jaceidin, 1‐O‐sinapoylglucose, ent‐epicatechin‐(4α→6)‐ent‐epicatechin, trans‐caffeic acid [apiosyl‐(1→6)‐glucosyl] ester and edulisin IV, which were significantly increased after the KD (Figure 4 and Table S9). In the organoheterocyclic compounds class, pyridoxine was increased by KD consumption, whereas the abundance of 2(N)‐methyl‐norsalsolinol, indole‐3‐carboxylic acid, pyrophaeophorbide a, 3‐formyl‐6‐hydroxyindole, and 6,7‐dihydro‐2,5‐dimethyl‐5H‐cyclopentapyrazine was decreased (Figure 4 and Table S9). We also identified several other metabolites as natural products, which were mainly found in insects, microbes, and herbal medicine plants, such as organic oxygen compounds (melezitose, D‐maltose, and D‐mannose), organooxygen compounds (ovalicin), and organohalogen compounds (ginsenoyne B) (Figure 4 and Table S9).

### Microbial pathway and metabolic associations in patients with WS after KD consumption

**FIGURE 5:**
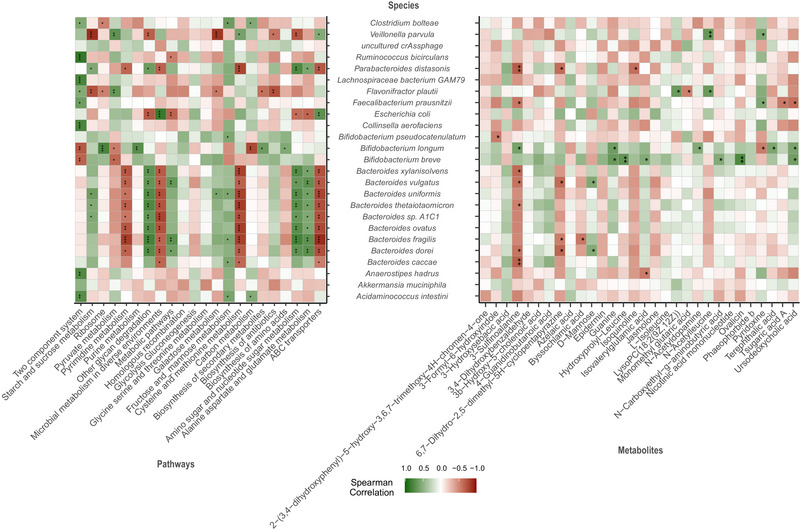
Correlation heatmap of significantly altered bacteria, KEGG pathways, and metabolites. This heatmap illustrates the Spearman's rank correlation (ρ) among significantly changed bacterial species, KEGG functional pathways, and metabolites between Pre_KD and Post_KD groups. Correlations were calculated using Spearman's rank correlation coefficient, with *P*‐values adjusted for multiple testing using the false discovery rate (FDR); *P < 0.05, **P < 0.01, ***P < 0.001 (FDR‐adjusted). The color gradient represents the strength and direction of correlation: green (positive), white (no correlation), and red (negative), ranging from +1.0 to −1.0. Only features that showed significant changes in abundance (via Wilcoxon signed‐rank test, FDR‐adjusted *P* < 0.05) were included in this analysis. Pre_KD, samples collected before ketogenic diet initiation; Post_KD, samples collected after 1 week of ketogenic diet therapy.

*Bacteroides* and *Parabacteroides* species showed significant correlations with certain KEGG pathways, exhibiting negative associations with ABC transporters, cysteine and methionine metabolism, microbial metabolism in diverse environments, and pyrimidine metabolism. In contrast, they showed positive associations with alanine aspartate and glutamate metabolism, amino sugar and nucleotide sugar metabolism, and other glycan degradation (Figure 5). However, *E. coli* showed an opposite pattern. *B. longum* was negatively associated with the two‐component system, pyruvate metabolism and carbon metabolism; Conversely, it was positively associated with ribosome, purine metabolism, biosynthesis of secondary metabolites, and biosynthesis of amino acids (Figure 5). There is also an interrelationship between GM and Metabolites. *Bacteroides* species and *P. distasonis* were negatively correlated with 3‐sulfinoalanine, 6,7‐dihydro‐2,5‐dimethyl‐5H‐cyclopentapyrazine. Conversely, *B. longum* was positively correlated with 3‐sulfinoalanine. *B. breve* was positively correlated with guanine, hydroxyprolyl‐leucine, isovalerylglutamic acid, N‐carboxyethyl‐g‐aminobutyric acid, ovalicin, and ursodeoxycholic acid (Figure 5).

## DISCUSSION

The etiology of WS is complex, and the complex interplay between KD consumption and the treatment of WS has yielded profound insights into the intricate relationships among microbial communities, metabolite profiles, and therapeutic responses. The GM plays a pivotal role in host health, regulating behavior, immune responses, and metabolic and nervous system activity through the ‘microbiota‐gut‐brain axis’.^24^, ^25^ This mutual communication implies that specific diets can potentially reconfigure the GM, and the GM can reciprocally influence diet efficacy.^26^, ^27^, ^28^ KD interventions rapidly shift the host's energy source to ketones, altering the nutritional substrate of gut microbes and various antiepileptic microbial metabolites.^18^ In 10 of 16 patients with WS in this study, the frequency of seizures decreased by more than 50% after 1 week of ketogenesis, highlighting that dietary intervention can quickly alleviate seizures, consistent with previous findings.^7^, ^8^

In this study, we utilized metagenome technology to construct a comprehensive GM profile of patients with WS, revealing uncharacterized but health‐related organisms.^29^, ^30^ PERMANOVA demonstrated that GM variation is strongly associated with the disease status, with distinct clustering of GM compositions observed in PCA, as reported previously.^31^ *Bacteroides*, *Bifidobacterium*, *Parabacteroides*, and *Escherichia* were found to be significant components of the GM. Compared to healthy controls, the abundance of *Proteobacteria* was higher, and that of *Bacteroidetes* was lower in patients with WS.^32^ *Bacteroidetes* species play a role in maintaining homeostasis in early life.^33^ *B. longum* and *B. breve*, which are enriched in patients with drug‐resistant epilepsy, are associated with higher levels of TNF, a pro‐inflammatory cytokine implicated in epilepsy induction.^34^ Interestingly, the higher abundance of both *B. longum* and *B. breve* corresponded to a decreased seizure frequency compared to the findings in patients who did not respond to KD consumption.^34^ The increased abundance of *B. fragilis* and pathogenic *E. coli* was positively associated with the onset of epilepsy in adults.^35^ In contrast, another study found that *B. fragilis* strain BF839 was beneficial for the treatment of refractory epilepsy (with study participants including both children and adults).^36^ This suggests that gut microbiota may exert different effects in different populations, and distinct strains may also play varying roles. Further clinical studies with larger sample sizes are required to resolve these controversial issues. In our study, the median abundance of *B. fragilis* showed an 8.79‐fold increase after KD, although it was not statistically significant, possibly due to the small sample size. After KD consumption, the abundance of several pathogens tended to decrease, including *S. enterica* and *E. coli*. Olson et al.^15^ identified *A. muciniphila* and *Parabacteroides* as two key microbes influencing the outcome of KD feeding in epileptic mice. These microbes were also enriched in patients with WS after KD therapy. These changes might potentially impact the host's metabolic state and the mechanisms underlying epilepsy, either directly or indirectly. Such observations underscore the potential significance of specific microbial taxa in patients with WS, further supporting the need to explore their potential roles in disease etiology and treatment responses.

An imbalance of GM leads to altered function. KEGG pathway analysis revealed significant alterations in vital metabolic pathways, particularly those involved in material transportation, energy production, and redox balance. After the intervention of KD, ABC transporters, glycine serine and threonine metabolism, oxocarboxylic acid metabolism, fatty acid biosynthesis, glutathione metabolism, tyrosine metabolism, and C5‐branched dibasic acid metabolism recovered. The ABC transporter is a large family. It mainly includes P‐glycoprotein (P‐gp), breast cancer resistance protein (BCRP), and multidrug resistance protein (MRP). Mechanistic and molecular studies implicate P‐gp as being involved in the acquisition of drug‐resistant epilepsy.^37^ The overexpression of efflux transporters in the blood‐brain barrier, mainly those from the ABC superfamily, might be responsible for limiting the accumulation of AEDs in the brain.^38^ In our study, ABC transporters were significantly downregulated after KD, which might enhance the efficacy of AEDs. However, further research is needed. Oxocarboxylic acid includes oxaloacetate, α‐ketoglutarate, pyruvate, etc., which are key molecules in the tricarboxylic acid cycle (TCA cycle). Tyrosine is a precursor of neurotransmitters and can effectively increase the levels of neurotransmitters in plasma, especially dopamine and norepinephrine, which have the functions of regulating mood, movement, and cognition.^39^, ^40^ And the dopaminergic system plays an important role in the regulation of epileptic seizures.^41^

The involvement of microbial metabolism in diverse pathways encompasses various metabolite biosynthesis and metabolism, such as butyrate synthesis from the butyryl‐CoA reaction.^42^ The decreases in neurotransmitter levels after KD consumption, including tryptamine, serotonin, and dopamine, provide intriguing evidence of its impact on neurochemical pathways.^43^ In addition, several new antiepileptic therapies targeting metabolic pathways have been proposed, such as inhibiting glycolysis by targeting lactate dehydrogenase, thereby decreasing intracellular ATP concentrations and suppressing neuronal excitation.^44^

Restoration of the GM structure, coupled with shifts in metabolite profiles, highlights the dynamic nature of the microbiota‐gut‐brain axis and its influence on disease‐specific microbial metabolite levels.^24^, ^45^ Glutamate, a principal excitatory neurotransmitter, is implicated in seizure initiation and spread,^44^ whereas lysine can prolong seizure latency by modulating GABAergic transmission.^46^ Metabolites synthesized by the GM and utilized by the host regulate the central nervous system and impact brain function.^47^ Biogenic neurotransmitters (e.g., γ‐aminobutyric acid [GABA] and serotonin) and metabolites (e.g., short‐chain fatty acids) play roles in relieving stress and promoting intestinal nervous system maturation.^48^, ^49^ Increased levels of curcumin III, capric acid, jaceidin, pyridoxine, and (3R)‐3,4‐dihydroxy‐3‐(hydroxymethyl)butanenitrile 4‐glucoside, pyridoxine are linked to anticonvulsant effects. Curcumin has antioxidant functions. Kumar et al.^50^ found that epileptic rats fed with curcumin showed a significant decrease in epileptiform activity. Capric acid is a 10‐carbon medium‐chain fatty acid, showed anticonvulsant efficacy in seizure tests in mice.^51^ Jaceidin is a type of flavonoid compound. Flavonoids show potential efficacy in the treatment of epilepsy. Certain flavonoids exhibit significant synergistic effects with AEDs, which help reduce the possibility of drug resistance. This may be related to the regulation of GABA receptors.^52^ Pyridoxine is involved in the metabolic process of neurotransmitters, including GABA and glycine. A deficiency of pyridoxal 5'‐phosphate (PLP) can induce epilepsy, and treatment with pyridoxal is effective.^53^ Reduced levels of N‐succinyl‐L,L‐2,6‐diaminopimelate, primarily metabolized by *E. coli*, might affect the TCA cycle. 4‐hydroxybenzaldehyde is a major active compound in Gastrodiae Rhizoma, an important Chinese herbal medicine used to manage dizziness and epilepsy.^54^ However, sucralose levels were significantly increased by KD consumption despite its known association with epilepsy.^55^ This bidirectional modulation of metabolites underscores the multifaceted roles of metabolites in the response to KD therapy.^56^ This intricate response might contribute to the observed clinical improvements in responders.

Furthermore, our correlation analyses revealed intricate relationships among microbes, KEGG pathways, and metabolites, creating a more comprehensive understanding of the KD–GM relationship. Certain key species, such as *Bacteroides* and *Parabacteroides*, exhibited strong associations with ABC transporters. ABC transporters are involved in the active pumping of many different substrates across cell membranes and regulate the pharmacokinetics of numerous drugs. The overexpression of certain transporters has been identified as a key factor in developing resistance to chemotherapeutic agents. ABC transporters have emerged as potential therapeutic targets for overcoming multidrug resistance.^57^, ^58^, ^59^, ^60^ This may be associated with the resistance mechanism of AEDs. The KD might downregulate ABC transporters by modulating GM composition, thereby mitigating AED resistance. However, further mechanistic investigations are required to validate this hypothesis. 3‐Sulfinoalanine, also called cysteine sulfinic acid (CSA), is converted to taurine by the catalytic action of CSA decarboxylase. In our study, 3‐sulfinoalanine significantly decreased after KD. *Bacteroides* species and *P. distasonis* were negatively correlated with 3‐sulfinoalanine, whereas *B. longum* showed a positive correlation with it. CSA acts as an agonist of metabotropic glutamate receptors (mGluRs), participating in neurotransmission through the phospholipase D pathway.^61^ Agonists of mGluRs are closely associated with the pathogenesis of epilepsy.^62^ However, studies have shown that taurine, an effective inhibitory neuromodulator, exerts antiepileptic effects and represents a potential therapeutic target for epilepsy. Next, the activity of cysteine sulfinate decarboxylase and the level of taurine can be further detected.^63^, ^64^, ^65^

Numerous studies have indicated that GMs are involved in the anti‐epileptic effects of the KD.^66^ However, current findings are inconsistent. Our study found that multiple KEGG pathways underwent significant changes after KD and tended to approach the levels of normal healthy individuals, including glycine, serine, and threonine metabolism, fatty acid biosynthesis, arginine and proline metabolism, glutathione metabolism, tyrosine metabolism, glycosaminoglycan degradation, and glycosphingolipid biosynthesis (globo series). Lum et al.^67^ transferred gut microbiota from children with refractory epilepsy to germ‐free mice and demonstrated that the post‐KD microbiota conferred seizure protection in recipient animals. Metabolomic profiling revealed that certain microbial pathways — such as those related to amino acid metabolism — were enriched in patients after KD treatment. Notably, while functional shifts were consistent, there was no universal taxonomic signature across individuals, potentially due to variations in epilepsy types and KD formulations.^67^ Özcan et al.^68^ found that dietary fiber plays an important role in the anti‐epileptic effects of the KD, with increased α‐diversity observed in the fiber‐supplemented KD group. Notably, both that study and our present work show a notable decrease in *Actinobacteria* and a marked increase in *Bacteroidetes* following KD. However, the changes in metagenomic pathways differed between the two studies, potentially due to host‐specific factors.^68^ A study by Lindefeldt et al.^69^ reported reduced *Bifidobacterium* and increased *E. coli* after 3 months of KD in children with therapy‐resistant epilepsy, with functional shifts primarily in the carbohydrate metabolism pathway. In contrast, our cohort showed a trend toward decreased *E. coli* and more prominent changes in amino acid and lipid metabolism after just 1 week of KD. These differences may reflect the shorter intervention period and distinct control group design. Wang et al.^70^ reported a significant increase in Bacteroides and its subspecies *B. fragilis* following KD in children with mitochondrial epilepsy. They also observed enrichment in metabolic pathways such as lysosome and glycosphingolipid biosynthesis, along with reduced activity in nicotinate and nicotinamide metabolism and ABC transporters. Our findings are largely consistent with these results, supporting a shared microbial and functional response to KD across different patient populations. In a mouse model of infantile spasms, Mu et al.^71^ reported increased gut microbiota alpha diversity, reduced *E. coli* abundance, and elevated microbial gene expression in phenylalanine, tyrosine, and tryptophan metabolism following KD treatment. These functional shifts parallel our findings, suggesting conserved metabolic adaptations in the GM during KD therapy.

The advantages of our study are as follows. Our research population consists of patients with the same type of epilepsy, all being cases of WS. WS shows a good response to KD. Patients are concentrated in the infant and toddler age group, and their unique seizure patterns are easy to observe. Furthermore, our study included healthy children of a similar age as controls. Several limitations of this study should be acknowledged. First, the sample size was small, which limited our ability to perform subgroup analyses—particularly between dietary responders and non‐responders—and reduced the statistical power to detect subtle but biologically relevant changes. As a result, we could not robustly assess which specific microbiome or metabolite alterations were directly associated with seizure improvement. Additionally, the limited cohort size hampers evaluation of potential confounding factors, such as concomitant AEDs, genetic backgrounds, and individual variations in gut microbiota composition.^72^ While correlation analyses provide valuable insights into potential interactions, they do not confirm functional capacity. Future studies integrating metatranscriptomics or culturomics are essential to validate whether the identified taxa are indeed responsible for the observed metabolic activities.

In conclusion, our study highlighted significant alterations in the GM composition and metabolites following KD therapy in patients with WS. These changes associated with the KD may contribute to seizure improvement.

## CONFLICT OF INTEREST

The authors declare no conflict of interest.

## Supporting information



Supporting Information

## References

[1] Devinsky O , Vezzani A , O'Brien TJ , Jette N , Scheffer IE , Curtis M , et al. Epilepsy. Nat Rev Dis Primers. 2018;4:18024. DOI: 10.1038/nrdp.2018.24 29722352

[2] Knight E , Wyllie E . West Syndrome and the new classification of epilepsy. Lancet Neurol. 2022;21:689. DOI: 10.1016/S1474-4422(22)00267-8 35841906

[3] Pavone P , Polizzi A , Marino SD , Corsello G , Falsaperla R , Marino S , et al. West syndrome: a comprehensive review. Neurol Sci. 2020;41:3547‐3562. DOI: 10.1007/s10072-020-04600-5 32827285 PMC7655587

[4] Choudhary A , Mu C , Barrett KT , Charkhand B , Williams‐Dyjur C , Marks WN , et al. The link between brain acidosis, breathing and seizures: a novel mechanism of action for the ketogenic diet in a model of infantile spasms. Brain Commun. 2021;3:fcab189. DOI: 10.1093/braincomms/fcab189 34734183 PMC8557655

[5] Song JM , Hahn J , Kim SH , Chang MJ . Efficacy of treatments for infantile spasms: a systematic review. Clin Neuropharmacol. 2017;40:63‐84. DOI: 10.1097/WNF.0000000000000200 28288483

[6] Ruan Y , Chen L , She D , Chung Y , Ge L , Han L . Ketogenic diet for epilepsy: an overview of systematic review and meta‐analysis. Eur J Clin Nutr. 2022;76:1234‐1244. DOI: 10.1038/s41430-021-01060-8 35027683

[7] Hong AM , Turner Z , Hamdy RF , Kossoff EH . Infantile spasms treated with the ketogenic diet: prospective single‐center experience in 104 consecutive infants. Epilepsia. 2010;51:1403‐1407. DOI: 10.1111/j.1528-1167.2010.02586.x 20477843

[8] Kossoff EH , Laux LC , Blackford R , Morrison PF , Pyzik PL , Hamdy RM , et al. When do seizures usually improve with the ketogenic diet? Epilepsia. 2008;49:329‐333. DOI: 10.1111/j.1528-1167.2007.01417.x 18028405

[9] Prezioso G , Carlone G , Zaccara G , Verrotti A . Efficacy of ketogenic diet for infantile spasms: a systematic review. Acta Neurol Scand. 2018;137:4‐11. DOI: 10.1111/ane.12830 28875525

[10] Kossoff EH , Zupec‐Kania BA , Auvin S , Ballaban‐Gil KR , Christina Bergqvist AG , Blackford R , et al. Optimal clinical management of children receiving dietary therapies for epilepsy: updated recommendations of the International Ketogenic Diet Study Group. Epilepsia Open. 2018;3:175‐192. DOI: 10.1002/epi4.12225 29881797 PMC5983110

[11] Devi N , Madaan P , Kandoth N , Bansal D , Sahu JK . Efficacy and safety of dietary therapies for childhood drug‐resistant epilepsy: a systematic review and network meta‐analysis. JAMA Pediatr. 2023;177:258‐266. DOI: 10.1001/jamapediatrics.2022.5648 36716045 PMC9887534

[12] David LA , Maurice CF , Carmody RN , Gootenberg DB , Button JE , Wolfe BE , et al. Diet rapidly and reproducibly alters the human gut microbiome. Nature. 2014;505:559‐563. DOI: 10.1038/nature12820 24336217 PMC3957428

[13] Zhang Y , Zhou S , Zhou Y , Yu L , Zhang L , Wang Y . Altered gut microbiome composition in children with refractory epilepsy after ketogenic diet. Epilepsy Res. 2018;145:163‐168. DOI: 10.1016/j.eplepsyres.2018.06.015 30007242

[14] Xie G , Zhou Q , Qiu CZ , Dai WK , Wang HP , Li YH , et al. Ketogenic diet poses a significant effect on imbalanced gut microbiota in infants with refractory epilepsy. World J Gastroenterol. 2017;23:6164‐6171. DOI: 10.3748/wjg.v23.i33.6164 28970732 PMC5597508

[15] Olson CA , Vuong HE , Yano JM , Liang QY , Nusbaum DJ , Hsiao EY . The gut microbiota mediates the anti‐seizure effects of the ketogenic diet. Cell. 2018;173:1728‐1741. DOI: 10.1016/j.cell.2018.04.027. e13. 29804833 PMC6003870

[16] Santangelo A , Corsello A , Spolidoro GCI , Trovato CM , Agostoni C , Orsini A , et al. The influence of ketogenic diet on gut microbiota: potential benefits, risks and indications. Nutrients. 2023;15:3680. DOI: 10.3390/nu15173680 37686712 PMC10489661

[17] Dyńka D , Kowalcze K , Paziewska A . The role of ketogenic diet in the treatment of neurological diseases. Nutrients. 2022;14:5003. DOI: 10.3390/nu14235003 36501033 PMC9739023

[18] Dahlin M , Prast‐Nielsen S . The gut microbiome and epilepsy. EBioMedicine. 2019;44:741‐746. DOI: 10.1016/j.ebiom.2019.05.024 31160269 PMC6604367

[19] Sorboni SG , Moghaddam HS , Jafarzadeh‐Esfehani R , Soleimanpour S . A comprehensive review on the role of the gut microbiome in human neurological disorders. Clin Microbiol Rev. 2022;35:e0033820. DOI: 10.1128/CMR.00338-20 34985325 PMC8729913

[20] Berg AT , Berkovic SF , Brodie MJ , Buchhalter J , Cross JH , van Emde Boas W , et al. Revised terminology and concepts for organization of seizures and epilepsies: report of the ILAE Commission on Classification and Terminology, 2005‐2009. Epilepsia. 2010;51:676‐685. DOI: 10.1111/j.1528-1167.2010.02522.x 20196795

[21] Li D , Li Y , Dai W , Wang H , Qiu C , Feng S , et al. Intestinal *Bacteroides* sp. imbalance associated with the occurrence of childhood undernutrition in China. Front Microbiol. 2019;10:2635. DOI: 10.3389/fmicb.2019.02635 31849851 PMC6895006

[22] Wood DE , Lu J , Langmead B . Improved metagenomic analysis with Kraken 2. Genome Biol. 2019;20:257. DOI: 10.1186/s13059-019-1891-0 31779668 PMC6883579

[23] Xie H , Guo R , Zhong H , Feng Q , Lan Z , Qin B , et al. Shotgun metagenomics of 250 adult twins reveals genetic and environmental impacts on the gut microbiome. Cell Syst. 2016;3:572‐584. DOI: 10.1016/j.cels.2016.10.004. e3. 27818083 PMC6309625

[24] Quigley EMM . Microbiota‐brain‐gut axis and neurodegenerative diseases. Curr Neurol Neurosci Rep. 2017;17:94. DOI: 10.1007/s11910-017-0802-6 29039142

[25] Ding M , Lang Y , Shu H , Shao J , Cui L . Microbiota‐gut‐brain axis and epilepsy: a review on mechanisms and potential therapeutics. Front Immunol. 2021;12:742449. DOI: 10.3389/fimmu.2021.742449 34707612 PMC8542678

[26] Illiano P , Brambilla R , Parolini C . The mutual interplay of gut microbiota, diet and human disease. FEBS J. 2020;287:833‐855. DOI: 10.1111/febs.15217 31955527

[27] Hey G , Nair N , Klann E , Gurrala A , Safarpour D , Mai V , et al. Therapies for Parkinson's disease and the gut microbiome: evidence for bidirectional connection. Front Aging Neurosci. 2023;15:1151850. DOI: 10.3389/fnagi.2023.1151850 37323145 PMC10261989

[28] Moszak M , Szulińska M , Bogdański P . You are what you eat–The relationship between diet, microbiota, and metabolic disorders–a review. Nutrients. 2020;12:1096. DOI: 10.3390/nu12041096 32326604 PMC7230850

[29] de Cena JA , Zhang J , Deng D , Damé‐Teixeira N , Do T . Low‐abundant microorganisms: the human microbiome's dark matter, a scoping review. Front Cell Infect Microbiol. 2021;11:689197. DOI: 10.3389/fcimb.2021.689197 34136418 PMC8201079

[30] Zha Y , Chong H , Yang P , Ning K . Microbial dark matter: from discovery to applications. Genomics Proteomics Bioinformatics. 2022;20:867‐881. DOI: 10.1016/j.gpb.2022.02.007 35477055 PMC10025686

[31] Xu L , Chen D , Zhao C , Jiang L , Mao S , Song C , et al. Decreased abundance of *Akkermansia* after adrenocorticotropic hormone therapy in patients with West syndrome. BMC Microbiol. 2021;21:126. DOI: 10.1186/s12866-021-02189-z 33892634 PMC8063292

[32] Şafak B , Altunan B , Topçu B , Eren Topkaya A . The gut microbiome in epilepsy. Microb Pathog. 2020;139:103853. DOI: 10.1016/j.micpath.2019.103853 31730997

[33] Rodríguez JM , Murphy K , Stanton C , Ross RP , Kober OI , Juge N , et al. The composition of the gut microbiota throughout life, with an emphasis on early life. Microb Ecol Health Dis. 2015;26:26050. DOI: 10.3402/mehd.v26.26050 25651996 PMC4315782

[34] Dahlin M , Singleton SS , David JA , Basuchoudhary A , Wickström R , Mazumder R , et al. Higher levels of *Bifidobacteria* and tumor necrosis factor in children with drug‐resistant epilepsy are associated with anti‐seizure response to the ketogenic diet. EBioMedicine. 2022;80:104061. DOI: 10.1016/j.ebiom.2022.104061 35598439 PMC9126955

[35] Dong L , Zheng Q , Cheng Y , Zhou M , Wang M , Xu J , et al. Gut microbial characteristics of adult patients with epilepsy. Front Neurosci. 2022;16:803538. DOI: 10.3389/fnins.2022.803538 35250450 PMC8888681

[36] Deng Y , Lin C , Cao D . The beneficial effect of *Bacteroides Fragilis* (BF839) as a supplementary treatment in drug‐resistant epilepsy: a pilot study (in Chinese). J Epilepsy. 2021;7:288‐295. DOI: 10.7507/2096-0247.20210046

[37] Heinrich A , Zhong XB , Rasmussen TP . Variability in expression of the human MDR1 drug efflux transporter and genetic variation of the *ABCB1* gene: implications for drug‐resistant epilepsy. Curr Opin Toxicol. 2018;11‐12:35‐42. DOI: 10.1016/j.cotox.2018.12.004 PMC6785833 31602418

[38] Leandro K , Bicker J , Alves G , Falcão A , Fortuna A . ABC transporters in drug‐resistant epilepsy: mechanisms of upregulation and therapeutic approaches. Pharmacol Res. 2019;144:357‐376. DOI: 10.1016/j.phrs.2019.04.031 31051235

[39] Daubner SC , Le T , Wang S . Tyrosine hydroxylase and regulation of dopamine synthesis. Arch Biochem Biophys. 2011;508:1‐12. DOI: 10.1016/j.abb.2010.12.017 21176768 PMC3065393

[40] Tarazi FI . Neuropharmacology of dopamine receptors: implications in neuropsychiatric diseases. J Sci Res Med Sci. 2001;3:93‐104. 24019715 PMC3174705

[41] Akyuz E , Polat AK , Eroglu E , Kullu I , Angelopoulou E , Paudel YN . Revisiting the role of neurotransmitters in epilepsy: an updated review. Life Sci. 2021;265:118826. DOI: 10.1016/j.lfs.2020.118826 33259863

[42] Yang Q , Guo S , Lu Q , Tao Y , Zheng D , Zhou Q , et al. Butyryl/Caproyl‐CoA:acetate CoA‐transferase: cloning, expression and characterization of the key enzyme involved in medium‐chain fatty acid biosynthesis. Biosci Rep. 2021;41:BSR20211135. DOI: 10.1042/BSR20211135 34338280 PMC8360832

[43] Operto FF , Matricardi S , Pastorino GMG , Verrotti A , Coppola G . The ketogenic diet for the treatment of mood disorders in comorbidity with epilepsy in children and adolescents. Front Pharmacol. 2020;11:578396. DOI: 10.3389/fphar.2020.578396 33381032 PMC7768824

[44] Sada N , Suto S , Suzuki M , Usui S , Inoue T . Upregulation of lactate dehydrogenase A in a chronic model of temporal lobe epilepsy. Epilepsia. 2020;61:e37‐e42. DOI: 10.1111/epi.16488 32202309

[45] Liang S , Wu X , Hu X , Wang T , Jin F . Recognizing depression from the microbiota‐gut‐brain axis. Int J Mol Sci. 2018;19:1592. DOI: 10.3390/ijms19061592 29843470 PMC6032096

[46] Ebrahimi HA , Ebrahimi S . Evaluation of the effects of charged amino acids on uncontrolled seizures. Neurol Res Int. 2015;2015:124507. DOI: 10.1155/2015/124507 26240759 PMC4512581

[47] Khatibi VA , Salimi M , Rahdar M , Rezaei M , Nazari M , Dehghan S , et al. Glycolysis inhibition partially resets epilepsy‐induced alterations in the dorsal hippocampus‐basolateral amygdala circuit involved in anxiety‐like behavior. Sci Rep. 2023;13:6520. DOI: 10.1038/s41598-023-33710-1 37085688 PMC10119516

[48] van de Wouw M , Boehme M , Lyte JM , Wiley N , Strain C , O'Sullivan O , et al. Short‐chain fatty acids: microbial metabolites that alleviate stress‐induced brain‐gut axis alterations. J Physiol. 2018;596:4923‐4944. DOI: 10.1113/JP276431 30066368 PMC6187046

[49] De Vadder F , Grasset E , Mannerås Holm L , Karsenty G , Macpherson AJ , Olofsson LE , et al. Gut microbiota regulates maturation of the adult enteric nervous system via enteric serotonin networks. Proc Natl Acad Sci U S A. 2018;115:6458‐6463. DOI: 10.1073/pnas.1720017115 29866843 PMC6016808

[50] Kumar V , Prakash C , Singh R , Sharma D . Curcumin's antiepileptic effect, and alterations in Nav1.1 and Nav1.6 expression in iron‐induced epilepsy. Epilepsy Res. 2019;150:7‐16. DOI: 10.1016/j.eplepsyres.2018.12.007 30605865

[51] Wlaź P , Socała K , Nieoczym D , Żarnowski T , Żarnowska I , Czuczwar SJ , et al. Acute anticonvulsant effects of capric acid in seizure tests in mice. Prog Neuropsychopharmacol Biol Psychiatry. 2015;57:110‐116. DOI: 10.1016/j.pnpbp.2014.10.013 25445478

[52] Shrivastava A , Gupta JK , Goyal M . Flavonoids and antiepileptic drugs: a comprehensive review on their neuroprotective potentials. J Med Pharm Allied Sci. 2022;11:4179‐4186. DOI: 10.22270/jmpas.V11I1.2175

[53] Mastrangelo M , Cesario S . Update on the treatment of vitamin B6 dependent epilepsies. Expert Rev Neurother. 2019;19:1135‐1147. DOI: 10.1080/14737175.2019.1648212 31340680

[54] Liu Y , Lu YY , Huang L , Shi L , Zheng ZY , Chen JN , et al. Para‐hydroxybenzyl alcohol delays the progression of neurodegenerative diseases in models of *Caenorhabditis elegans* through activating multiple cellular protective pathways. Oxid Med Cell Longev. 2022;2022:8986287. DOI: 10.1155/2022/8986287 35401930 PMC8989581

[55] Galic MA , Persinger MA . Sucrose ingestion decreases seizure onset time in female rats treated with lithium and pilocarpine. Epilepsy Behav. 2005;6:552‐555. DOI: 10.1016/j.yebeh.2005.03.013 15907749

[56] Rawat K , Singh N , Kumari P , Saha L . A review on preventive role of ketogenic diet (KD) in CNS disorders from the gut microbiota perspective. Rev Neurosci. 2021;32:143‐157. DOI: 10.1515/revneuro-2020-0078 33070123

[57] Amawi H , Sim HM , Tiwari AK , Ambudkar SV , Shukla S . ABC transporter‐mediated multidrug‐resistant cancer. Adv Exp Med Biol. 2019;1141:549‐580. DOI: 10.1007/978-981-13-7647-4_12 31571174

[58] Bugde P , Biswas R , Merien F , Lu J , Liu DX , Chen M , et al. The therapeutic potential of targeting ABC transporters to combat multi‐drug resistance. Expert Opin Ther Targets. 2017;21:511‐530. DOI: 10.1080/14728222.2017.1310841 28335655

[59] Mohammadi F , Nejatollahi M , Sheikhnia F , Ebrahimi Y , Mohammadi M , Rashidi V , et al. MiRNAs: main players of cancer drug resistance target ABC transporters. Naunyn Schmiedebergs Arch Pharmacol. 2025;398:6239‐6291. DOI: 10.1007/s00210-024-03719-y 39808313

[60] Wu Y , Zhang J , Tian Y , Chi Shing Cho W , Xu HX , Lin ZX , et al. 20(*S*)‐Ginsenoside Rh2 overcomes gemcitabine resistance in pancreatic cancer by inhibiting LAMC2‐Modulated ABC transporters. J Adv Res. 2025;73:743‐760. DOI: 10.1016/j.jare.2024.09.006 39270979 PMC12225906

[61] Shi Q , Savage JE , Hufeisen SJ , Rauser L , Grajkowska E , Ernsberger P , et al. L‐homocysteine sulfinic acid and other acidic homocysteine derivatives are potent and selective metabotropic glutamate receptor agonists. J Pharmacol Exp Ther. 2003;305:131‐142. DOI: 10.1124/jpet.102.047092 12649361

[62] Huang L , Xiao W , Wang Y , Li J , Gong J , Tu E , et al. Metabotropic glutamate receptors (mGluRs) in epileptogenesis: an update on abnormal mGluRs signaling and its therapeutic implications. Neural Regen Res. 2024;19:360‐368. DOI: 10.4103/1673-5374.379018 37488891 PMC10503602

[63] Shukurova S , Sadek R , Mekawy N , Bendaoud M , Yachou Y , Mamchyn A , et al. Electrophysiological evidence for anti‐epileptic property of taurine. Adv Exp Med Biol. 2022;1370:333‐340. DOI: 10.1007/978-3-030-93337-1_32 35882808

[64] Jakaria M , Azam S , Haque ME , Jo SH , Uddin MS , Kim IS , et al. Taurine and its analogs in neurological disorders: focus on therapeutic potential and molecular mechanisms. Redox Biol. 2019;24:101223. DOI: 10.1016/j.redox.2019.101223 31141786 PMC6536745

[65] Jangra A , Gola P , Singh J , Gond P , Ghosh S , Rachamalla M , et al. Emergence of taurine as a therapeutic agent for neurological disorders. Neural Regen Res. 2024;19:62‐68. DOI: 10.4103/1673-5374.374139 37488845 PMC10479846

[66] Mu C , Rho JM , Shearer J . The interplay between the gut and ketogenic diets in health and disease. Adv Sci. 2025;12:e04249. DOI: 10.1002/advs.202504249 PMC12463008 40847749

[67] Lum GR , Ha SM , Olson CA , Blencowe M , Paramo J , Reyes B , et al. Ketogenic diet therapy for pediatric epilepsy is associated with alterations in the human gut microbiome that confer seizure resistance in mice. Cell Rep. 2023;42:113521. DOI: 10.1016/j.celrep.2023.113521 38070135 PMC10769314

[68] Özcan E , Yu KB , Dinh L , Lum GR , Lau K , Hsu J , et al. Dietary fiber content in clinical ketogenic diets modifies the gut microbiome and seizure resistance in mice. Nat Commun. 2025;16:987. DOI: 10.1038/s41467-025-56091-7 39856104 PMC11759687

[69] Lindefeldt M , Eng A , Darban H , Bjerkner A , Zetterström CK , Allander T , et al. The ketogenic diet influences taxonomic and functional composition of the gut microbiota in children with severe epilepsy. NPJ Biofilms Microbiomes. 2019;5:5. DOI: 10.1038/s41522-018-0073-2 30701077 PMC6344533

[70] Wang J , Huang L , Li H , Chen G , Yang L , Wang D , et al. Effects of ketogenic diet on the classification and functional composition of intestinal flora in children with mitochondrial epilepsy. Front Neurol. 2023;14:1237255. DOI: 10.3389/fneur.2023.1237255 37588668 PMC10426284

[71] Mu C , Choudhary A , Mayengbam S , Barrett KT , Rho JM , Shearer J , et al. Seizure modulation by the gut microbiota and tryptophan‐kynurenine metabolism in an animal model of infantile spasms. EBioMedicine. 2022;76:103833. DOI: 10.1016/j.ebiom.2022.103833 35090836 PMC8883001

[72] Helbig I , Tayoun AA . Understanding genotypes and phenotypes in epileptic encephalopathies. Mol Syndromol. 2016;7:172‐181. DOI: 10.1159/000448530 27781027 PMC5073622

