# Supplementary material for: The ketogenic diet alters microbiome‐metabolome profiles to improve West syndrome therapy
Source: Pediatr Investig. 2025 Nov 19;10(1):10–24. doi: 10.1002/ped4.70027 (PMC12921638; doi:10.1002/ped4.70027)
Supplement: Supplementary file 1 — Supporting Information [file PED4-10-10-s001.pdf]

Supplementary Material for

The ketogenic diet alters microbiome-metabolome profiles to improve West syndrome therapy

Gan Xie, Qian Zhou, Jianxiang Liao, Yuejie Zheng, Wenjian Wang, Kunling Shen

TABLE S1 Sample information

SubjectID	Group	Gender	Age (year)	Weight (kg)	Height (cm)	Side Effect	baseline seizure frequency (Pre-KD)	seizure frequency (Post-KD)	reduction in seizure frequency (%)	Drugs
H1	Healthy	Male	1.2	9.82	80	-				
H2	Healthy	Female	2.7	22	90	-				
H3	Healthy	Male	1.3	9.25	76	-				
H4	Healthy	Female	1.3	9.88	80	-				
H5	Healthy	Female	1.5	10.8	82	-				
H6	Healthy	Male	1.8	11.21	81	-				
H7	Healthy	Male	2.5	16.5	100	-				
H8	Healthy	Female	1	8.4	78	-				
H9	Healthy	Female	1.6	11	85.5	-				
H10	Healthy	Male	1.7	8.81	80.5	-				
H11	Healthy	Male	1.5	10.8	79	-				
H12	Healthy	Male	1.4	13.72	88	-				
H13	Healthy	Male	2.9	14.8	94.5	-				
H14	Healthy	Male	1.8	12.3	89	-				
H15	Healthy	Male	2.8	14.51	96	-				
H16	Healthy	Male	1.6	11.4	80	-				
H17	Healthy	Female	2.9	12.5	90	-				
H18	Healthy	Male	1.7	6.7	60	-				
H19	Healthy	Male	2.5	16.8	102	-				
H20	Healthy	Female	1.1	9.1	78	-				
H21	Healthy	Female	1.2	8.55	76	-				
H22	Healthy	Female	2.3	20	88	-				
H23	Healthy	Male	1.8	12	87	-				
H24	Healthy	Male	1.7	12	85.5	-				
P1	Epilepsy	Male	2.1	10.8	88	Vomit	3 times/Day	1 times/Day	50-90%	Levetiracetam, Sodium Valproate, Nitrazepam
P2	Epilepsy	Male	1.9	9.25	80	No	7.5 times/Day	3 times/Day	50-90%	Levetiracetam, Topiramate, Sodium Valproate, Nitrazepam, Valnoctamide, ACTH
P3	Epilepsy	Male	1	8.9	85	No	16.5 times/Day	16 times/Day	<25%	Levetiracetam, Sodium Valproate,

										Nitrazepam, Topiramate, ACTH
P4	Epilepsy	Male	1.1	9.3	80	No	4 times/Day	2 times/Day	50-90%	Levetiracetam, Nitrazepam, Topiramate
P5	Epilepsy	Male	1.3	11	82	Vomit	2.5 times/Day	0 times/Day	100%	Levetiracetam, Sodium Valproate
P6	Epilepsy	Female	1.3	7.5	76	No	15 times/Day	6 times/Day	50-90%	Levetiracetam, Topiramate, Nitrazepam, Phenobarbital, ACTH
P7	Epilepsy	Male	3.3	11.5	90	No	4.5 times/Day	2 times/Day	50-90%	Topiramate, Nitrazepam, Sodium Valproate, Valnoctamide, ACTH
P8	Epilepsy	Male	2.3	11.7	90	No	1 times/Day	1 times/Day	<25%	Levetiracetam, Sodium Valproate, Topiramate, ACTH, Clonazepam, Clonazepam
P9	Epilepsy	Female	1.1	6.8	74	Stomachache	4.5 times/Day	13 times/Day	<25%	Levetiracetam, Oxcarbazepine, Phenobarbital, Topiramate, Valnoctamide, Nitrazepam
P10	Epilepsy	Female	0.5	6.9	69	No	13 times/Day	0 times/Day	100%	Levetiracetam, ACTH
P11	Epilepsy	Male	0.7	8.7	71	No	2.5 times/Day	1 times/Day	50-90%	Levetiracetam, ACTH, Topiramate, Nitrazepam
P12	Epilepsy	Male	0.8	7.5	70	No	5.5 times/Day	4 times/Day	25-50%	Levetiracetam, Topiramate, Nitrazepam
P13	Epilepsy	Female	1	8	73	No	12 times/Day	11 times/Day	<25%	Levetiracetam, Lamotrigine,

										Oxcarbazepine, Sodium Valproate
P14	Epilepsy	Male	0.7	10.5	75	Diarrhea	5 times/Day	3 times/Day	25-50%	Phenobarbital, Lamotrigine, Sodium Valproate, Clonazepam
P15	Epilepsy	Male	1.7	11.8	84	No	20 times/Day	6 times/Day	50-90%	Levetiracetam, Sodium Valproate, Clonazepam, Topiramate, Prednisone
P16	Epilepsy	Female	2.5	28.6	92	No	4 times/Day	2 times/Day	50-90%	Sodium Valproate, Prednisone, Clonazepam

TABLE S2 Comparison between Healthy and Pre_KD groups at phylum level

Phylum	Healthy (median)	Health (Q1-Q3)	Pre_KD (median)	Pre_KD (Q1-Q3)	Healthy_vs_Pre_KD (p.value)	Healthy_vs_Pre_KD (p.adj)
**Bacteroidetes**	49.105	33.768-60.26	4.502	0.347-46.474	0.03423	0.1
**Unclassified**	29.73	25.615-44.062	20.782	10.843-33.693	0.05008	0.14
**Firmicutes**	9.23	7.018-14.08	9.454	4.943-20.379	0.82274	0.87
**Actinobacteria**	2.937	1.298-8.254	6.203	3.005-14.163	0.06668	0.1
**Proteobacteria**	1.171	0.877-1.754	17.587	3.351-31.182	0.00011	0.00034
**Chordata**	0.08	0.062-0.106	0.098	0.076-0.289	0.12762	0.19
**other**	0.034	0.022-0.047	0.022	0.012-0.041	0.15234	0.23
**Cyanobacteria**	0.019	0.014-0.028	0.012	0.008-0.025	0.08742	0.26
**Fusobacteria**	0.018	0.014-0.034	0.017	0.007-0.023	0.19059	0.29
**Euryarchaeota**	0.013	0.01-0.018	0.009	0.005-0.015	0.10222	0.29
**Verrucomicrobia**	0.012	0.008-0.028	0.007	0.004-0.013	0.12762	0.21
**Planctomycetes**	0.012	0.007-0.017	0.009	0.003-0.014	0.15234	0.34
**Spirochaetes**	0.011	0.009-0.019	0.008	0.005-0.015	0.18044	0.31
**Tenericutes**	0.008	0.007-0.013	0.007	0.004-0.019	0.4452	0.45

Pre_KD, samples collected before ketogenic diet initiation; Post_KD, samples collected after one week of ketogenic diet therapy.

TABLE S3 Comparison between Healthy and Pre_KD groups at genus level

Genus	Healthy (median)	Healthy (Q1-Q3)	Pre_KD (median)	Pre_KD (Q1-Q3)	Healthy_vs_Pre_KD (p.value)	Healthy_vs_Pre_KD (p.adj)
**Bacteroides**	39.86	28.56-52.151	3.295	0.132-44.974	0.04651	0.14
**Unclassified**	35.348	29.503-55.492	39.738	25.127-52.833	0.79957	0.99
**Bifidobacterium**	2.225	1.02-7.912	6.082	2.792-12.974	0.13549	0.14
**Faecalibacterium**	1.35	0.503-1.981	0.042	0.016-0.941	0.0123	0.018
**other**	0.841	0.577-1.198	0.701	0.384-0.988	0.32977	0.56
**Parabacteroides**	0.733	0.173-3.686	0.143	0.011-0.41	0.00764	0.023
**Lachnoclostridium**	0.495	0.312-0.872	0.817	0.47-1.262	0.22348	0.67
**Blautia**	0.442	0.312-0.549	0.33	0.124-0.774	0.37671	0.57
**Anaerostipes**	0.395	0.213-0.975	0.071	0.019-0.328	0.00928	0.028
**Flavonifractor**	0.297	0.129-0.449	1.012	0.401-1.61	0.02279	0.068
**Veillonella**	0.208	0.047-0.61	0.225	0.011-2.883	0.77657	0.98
**Roseburia**	0.184	0.105-0.482	0.065	0.024-0.227	0.02477	0.074
**Streptococcus**	0.183	0.13-0.265	0.17	0.101-0.269	0.96427	0.96
**Escherichia**	0.152	0.036-0.297	7.566	0.725-10.874	0.00015	0.00023
**Clostridioides**	0.143	0.103-0.185	0.148	0.083-0.199	0.84607	0.92
**Alistipes**	0.13	0.052-0.336	0.047	0.003-0.223	0.12762	0.38
**Ruthenibacterium**	0.129	0.031-0.297	0.043	0.012-0.175	0.12009	0.36
**Clostridium**	0.115	0.076-0.228	0.074	0.032-0.115	0.03998	0.12
**Anaerobutyricum**	0.102	0.061-0.157	0.018	0.012-0.065	0.0051	0.0085
**Eubacterium**	0.091	0.064-0.293	0.042	0.017-0.22	0.15234	0.46
**Paraprevotella**	0.078	0.052-0.12	0.005	0-0.033	0.00019	0.00056
**Butyricimonas**	0.049	0.026-0.081	0.005	0-0.012	0.00127	0.0038
**Prevotella**	0.046	0.023-0.094	0.012	0.005-0.025	0.00299	0.009
**Dysosmobacter**	0.044	0.021-0.071	0.047	0.016-0.081	0.79957	0.8
**Odoribacter**	0.044	0.014-0.109	0.009	0-0.027	0.00413	0.012
**Eggerthella**	0.04	0.027-0.104	0.085	0.034-0.222	0.18044	0.27
**Haemophilus**	0.039	0.017-0.171	0.003	0-0.025	0.00842	0.025
**Bacillus**	0.038	0.029-0.053	0.054	0.021-0.255	0.48187	0.72
**Mordavella**	0.037	0.028-0.063	0.02	0.005-0.038	0.02094	0.063
**Paenibacillus**	0.035	0.023-0.048	0.024	0.011-0.049	0.31498	0.39
**Barnesiella**	0.034	0.01-0.061	0.001	0.001-0.004	2.50E-05	7.50E-05
**Enterococcus**	0.03	0.021-0.053	0.079	0.03-0.161	0.0579	0.17
**Pseudomonas**	0.03	0.016-0.037	0.033	0.013-0.046	0.73115	0.73
**Lactobacillus**	0.027	0.016-0.033	0.025	0.013-0.034	0.52009	0.84
**Muribaculum**	0.025	0.01-0.049	0.003	0.001-0.006	0.00024	0.00071
**Klebsiella**	0.023	0.01-0.061	0.058	0.022-0.35	0.09953	0.3
**Ruminococcus**	0.021	0.012-0.045	0.013	0.004-0.112	0.26013	0.39
**Longibaculum**	0.02	0.012-0.032	0.008	0.002-0.023	0.05008	0.15
**Hungatella**	0.02	0.013-0.034	0.025	0.005-0.046	0.96427	0.96

**Intestinimonas**	0.019	0.015-0.042	0.016	0.007-0.035	0.37671	0.57
**Butyrivibrio**	0.019	0.014-0.029	0.01	0.005-0.021	0.03423	0.1
**Faecalitalea**	0.019	0.009-0.029	0.01	0.005-0.026	0.20114	0.3
**Christensenella**	0.017	0.01-0.023	0.013	0.006-0.028	0.53975	0.54
**Streptomyces**	0.017	0.012-0.022	0.012	0.005-0.024	0.36064	0.56
**Chryseobacterium**	0.017	0.011-0.029	0.008	0.004-0.013	0.00413	0.012
**Oscillibacter**	0.016	0.007-0.058	0.013	0.005-0.037	0.39321	0.39
**Fusobacterium**	0.013	0.008-0.021	0.01	0.003-0.015	0.17068	0.29
**Campylobacter**	0.012	0.008-0.022	0.011	0.003-0.021	0.27319	0.41
**Desulfovibrio**	0.011	0.005-0.023	0.006	0.001-0.049	0.42746	0.43
**Staphylococcus**	0.01	0.008-0.017	0.01	0.008-0.016	0.91675	0.92
**Enterobacter**	0.01	0.006-0.044	0.025	0.012-0.054	0.18044	0.29
Akkermansia	0.009	0.005-0.025	0.004	0.002-0.007	0.07649	0.23
**Flavobacterium**	0.009	0.007-0.017	0.004	0.003-0.006	0.00071	0.0021
**Corynebacterium**	0.008	0.005-0.011	0.009	0.004-0.013	0.91675	0.92
**Gordonibacter**	0.007	0.004-0.009	0.007	0.003-0.032	0.58013	0.82
**Duncaniella**	0.007	0.002-0.013	0.001	0-0.004	0.02061	0.062
**Collinsella**	0.007	0.004-0.457	0.004	0.001-0.011	0.08181	0.18
**Tannerella**	0.006	0.002-0.015	0.001	0-0.004	0.00928	0.028
**Monoglobus**	0.006	0.003-0.009	0.004	0.002-0.007	0.23527	0.6
**Megasphaera**	0.005	0.003-0.01	0.003	0.001-0.009	0.16132	0.48
**Citrobacter**	0.005	0.004-0.02	0.073	0.015-0.136	0.00118	0.0035
**Actinomyces**	0.005	0.004-0.007	0.017	0.008-0.033	0.00626	0.019
**Porphyromonas**	0.005	0.003-0.023	0.001	0-0.025	0.09953	0.3
**Sutterella**	0.004	0.001-0.027	0.001	0-0.003	0.01121	0.034
**Lactococcus**	0.004	0.003-0.005	0.006	0.003-0.054	0.07649	0.11
**Salmonella**	0.003	0.002-0.005	0.025	0.013-0.092	3.60E-05	0.00011
**Schaalia**	0.003	0.002-0.005	0.017	0.005-0.032	0.0051	0.015
**Acidaminococcus**	0.003	0.002-0.007	0.002	0.001-0.004	0.16132	0.31
**Phascolarctobacterium**	0.003	0.002-0.197	0.002	0.001-0.003	0.03998	0.12
**Rothia**	0.002	0.001-0.003	0.006	0.004-0.022	0.00169	0.0051
**Shigella**	0.002	0-0.004	0.024	0.011-0.117	0.00081	0.0024
**Morganella**	0.001	0.001-0.002	0.001	0.001-0.064	0.89309	0.89
**Megamonas**	0.001	0.001-0.002	0.001	0.001-0.003	0.82274	0.98
**Actinoalloteichus**	0	0-0.001	0.001	0-0.224	0.22608	0.34

Pre_KD, samples collected before ketogenic diet initiation.

TABLE S4 Comparison between Healthy and Pre_KD groups at species level

Species	Healthy (median)	Healthy (Q1-Q3)	Pre_KD (median)	Pre_KD (Q1-Q3)	Healthy_vs_Pre_KD (p.value)	Healthy_vs_Pre_KD (p.adj)
Unclassified	39.263	31.734-49.221	43.262	28.915-57.639	0.94049	0.94
Bacteroides_vulgatus	4.776	0.427-13.303	0.032	0.004-0.9	0.00169	0.0051
Bacteroides_fragilis	3.112	1.164-9.695	0.312	0.023-15.169	0.05008	0.15
other	1.361	1.106-1.986	1.585	1.039-1.895	0.77657	1
Faecalibacterium_prausnitzii	1.35	0.503-1.981	0.042	0.016-0.941	0.0123	0.018
Bacteroides_uniformis	0.85	0.281-5.384	0.1	0.006-0.279	0.00048	0.0014
Bacteroides_thetaiotaomicron	0.724	0.115-2.249	0.056	0.009-1.854	0.07649	0.23
Bacteroides_dorei	0.669	0.195-1.732	0.016	0.001-0.046	0.00019	0.00056
Bacteroides_sp._A1C1	0.534	0.162-1.702	0.025	0.006-0.125	0.00032	0.00095
Bifidobacterium_longum	0.495	0.087-1.263	0.509	0.11-2.654	0.66475	0.66
Parabacteroides_distasonis	0.416	0.08-2.426	0.077	0.007-0.182	0.00692	0.021
Anaerostipes_hadrus	0.377	0.118-0.924	0.022	0.007-0.058	0.00081	0.0024
X.Clostridium._bolteae	0.317	0.093-0.476	0.414	0.187-0.956	0.30062	0.9
Bifidobacterium_pseudocatenulatum	0.304	0.004-1.447	0.02	0.003-1.303	0.31498	0.62
Flavonifractor_plautii	0.297	0.129-0.449	1.012	0.401-1.61	0.02279	0.068
Bacteroides_ovatus	0.204	0.093-1.397	0.033	0.006-0.255	0.00764	0.023
Bacteroides_xylanisolvens	0.204	0.068-2.062	0.046	0.004-0.564	0.03998	0.12
Bacteroides_caecimuris	0.19	0.064-0.422	0.015	0.003-0.073	0.00118	0.0035
Blautia_sp._SC05B48	0.176	0.116-0.256	0.043	0.021-0.128	0.00626	0.019
Bifidobacterium_breve	0.165	0.052-0.809	1.118	0.448-3.414	0.00928	0.024
Lachnospiraceae_bacterium_Choco86	0.148	0.082-0.246	0.072	0.033-0.163	0.03162	0.095
Clostridioides_difficile	0.143	0.103-0.185	0.148	0.083-0.199	0.84607	0.92
Escherichia_coli	0.137	0.032-0.276	7.003	0.665-10.098	0.00015	0.00023
Ruthenibacterium_lactatiformans	0.129	0.031-0.297	0.043	0.012-0.175	0.12009	0.36
Veillonella_parvula	0.119	0.024-0.369	0.163	0.007-2.618	0.94049	0.95
Bacteroides_caccae	0.118	0.047-0.772	0.013	0.004-0.103	0.0123	0.037
Roseburia_intestinalis	0.104	0.065-0.205	0.035	0.013-0.087	0.00928	0.028
Anaerobutyricum_hallii	0.102	0.061-0.157	0.018	0.012-0.065	0.0051	0.0085
Bacteroides_intestinalis	0.101	0.028-0.198	0.008	0.001-0.024	0.00013	0.00039
Lachnospiraceae_bacterium_GAM79	0.094	0.053-0.245	0.039	0.011-0.084	0.01921	0.058
Blautia_hansenii	0.094	0.056-0.139	0.051	0.02-0.101	0.02094	0.063
Bacteroides_salanitronis	0.086	0.041-0.219	0.012	0.003-0.028	0.00032	0.00095
Roseburia_hominis	0.084	0.038-0.136	0.028	0.011-0.129	0.03701	0.11
Bacteroides_cellulosilyticus	0.082	0.044-0.181	0.011	0.001-0.025	3.00E-05	9.00E-05
Paraprevotella_xylaniphila	0.078	0.052-0.12	0.005	0-0.033	0.00019	0.00056
Lachnoclostridium_sp._YL32	0.075	0.047-0.087	0.074	0.026-0.226	0.75375	0.75
Clostridiales_bacterium_CCNA10	0.072	0.039-0.224	0.045	0.023-0.121	0.28669	0.64
X.Eubacterium._rectale	0.07	0.035-0.267	0.014	0.006-0.066	0.00413	0.012
Blautia_sp._N6H1_15	0.061	0.043-0.106	0.07	0.023-0.098	0.58013	0.87

**Bacteroides_helcogenes**	0.057	0.02-0.097	0.007	0.001-0.013	0.00032	0.00095
**Butyricimonas_faecalis**	0.049	0.026-0.081	0.005	0-0.012	0.00127	0.0038
**X.Clostridium._scindens**	0.049	0.036-0.073	0.041	0.011-0.061	0.2121	0.32
**X.Eubacterium._eligens**	0.044	0.021-0.266	0.009	0.003-0.035	0.00692	0.01
**Odoribacter_splanchnicus**	0.044	0.014-0.109	0.009	0-0.027	0.00413	0.012
**Dysosmobacter_welbionis**	0.044	0.021-0.071	0.047	0.016-0.081	0.79957	0.8
**Parabacteroides_sp._CT06**	0.043	0.019-0.08	0.008	0-0.026	0.00692	0.021
**Eggerthella_lenta**	0.04	0.026-0.104	0.083	0.034-0.22	0.20114	0.3
**Bacteroides_heparinolyticus**	0.04	0.011-0.083	0.004	0-0.006	0.00055	0.0016
**Mordavella_sp._Marseille_P3756**	0.037	0.028-0.063	0.02	0.005-0.038	0.02094	0.063
**Barnesiella_viscericola**	0.034	0.01-0.061	0.001	0.001-0.004	2.50E-05	7.50E-05
**Blautia_producta**	0.034	0.022-0.064	0.059	0.015-0.091	0.36064	0.36
**Haemophilus_parainfluenzae**	0.034	0.014-0.158	0.002	0-0.021	0.00764	0.023
**Bifidobacterium_catenulatum**	0.029	0.008-0.116	0.018	0.002-0.161	0.50079	0.6
**Lachnoclostridium_phocaeense**	0.029	0.022-0.051	0.032	0.009-0.047	0.66475	0.89
**Alistipes_finegoldii**	0.029	0.011-0.043	0.002	0.001-0.022	0.02477	0.074
**Veillonella_dispar**	0.029	0.013-0.113	0.051	0.002-0.156	0.64314	0.91
**Bacteroides_zoogleoformans**	0.025	0.008-0.036	0.003	0.001-0.007	0.00036	0.0011
**Lachnospiraceae_bacterium_KGMB03038**	0.024	0.013-0.042	0.026	0.008-0.038	0.70877	0.71
**Enterococcus_faecium**	0.021	0.011-0.033	0.022	0.013-0.045	0.64314	0.78
**Hungatella_hathewayi**	0.02	0.013-0.034	0.025	0.005-0.046	0.96427	0.96
**Longibaculum_sp._KGMB06250**	0.02	0.012-0.032	0.008	0.002-0.023	0.05008	0.15
**Bifidobacterium_bifidum**	0.019	0.003-0.078	0.034	0.002-0.072	0.98809	0.99
**Faecalitalea_cylindroides**	0.019	0.009-0.029	0.01	0.005-0.026	0.20114	0.3
**Intestinimonas_butyriciproducens**	0.019	0.015-0.042	0.016	0.007-0.035	0.37671	0.57
**Anaerostipes_rhamnosivorans**	0.017	0.01-0.038	0.021	0.013-0.063	0.797	0.8
**Clostridium_sporogenes**	0.017	0.007-0.028	0.003	0.001-0.012	0.00371	0.011
**Streptococcus_salivarius**	0.014	0.005-0.032	0.013	0.003-0.042	0.75375	0.75
**Oscillibacter_sp._PEA192**	0.014	0.004-0.048	0.009	0.003-0.026	0.46334	0.46
**Prevotella_intermedia**	0.013	0.006-0.026	0.003	0-0.007	0.00299	0.009
**X.Clostridium._saccharolyticum**	0.013	0.009-0.021	0.017	0.004-0.031	1	1
**Erysipelotrichaceae_bacterium_GAM147**	0.012	0.007-0.024	0.008	0.004-0.011	0.08181	0.12
**X.Clostridium._sphenoides**	0.011	0.009-0.019	0.018	0.005-0.031	0.77657	0.87
**Ruminococcus_bicirculans**	0.01	0.005-0.024	0.002	0.001-0.046	0.09332	0.14
**Streptococcus_parasanguinis**	0.01	0.005-0.015	0.012	0.003-0.025	0.84607	0.85
**Bifidobacterium_adolescentis**	0.01	0.005-0.028	0.009	0.001-0.032	0.53975	0.7
**Clostridium_sp._SY8519**	0.009	0.006-0.014	0.007	0.003-0.013	0.26013	0.49
**Alistipes_sp._6CPBBH3**	0.009	0.002-0.021	0.001	0-0.007	0.02232	0.067
**Alistipes_sp._Marseille_P5997**	0.009	0.002-0.038	0.001	0-0.003	0.00103	0.0031
**Akkermansia_muciniphila**	0.008	0.005-0.023	0.004	0.002-0.007	0.08181	0.25
**Streptococcus_mitis**	0.008	0.004-0.019	0.008	0.004-0.011	0.75375	0.75
**Alistipes_shahii**	0.008	0.004-0.016	0.002	0.001-0.011	0.0579	0.17

**Streptococcus_pasteurianus**	0.008	0.005-0.021	0.008	0.002-0.015	0.31498	0.47
**Collinsella_aerofaciens**	0.007	0.004-0.457	0.004	0.001-0.011	0.08181	0.18
**Streptococcus_sp._HSISM1**	0.007	0.005-0.012	0.009	0.003-0.02	0.96427	0.96
**Bacteroides_coprosuis**	0.007	0.003-0.015	0.001	0-0.001	0.00055	0.0016
**Clostridium_perfringens**	0.007	0.004-0.022	0.006	0.002-0.008	0.24749	0.45
**Eubacterium_limosum**	0.007	0.005-0.012	0.01	0.003-0.032	1	1
**X.Eubacterium._cellulosolvens**	0.007	0.006-0.011	0.006	0.001-0.01	0.27319	0.51
**Monoglobus_pectinilyticus**	0.006	0.003-0.009	0.004	0.002-0.007	0.23527	0.6
**Clostridium_botulinum**	0.006	0.005-0.01	0.005	0.002-0.008	0.2121	0.54
**Christensenella_minuta**	0.005	0.004-0.015	0.005	0.002-0.015	0.41013	0.41
**Eubacterium_maltosivorans**	0.005	0.004-0.008	0.006	0.002-0.01	0.62183	0.62
**Ruminococcus_sp._JE7A12**	0.005	0.003-0.007	0.002	0.001-0.01	0.18044	0.27
**Alistipes_sp._5CBH24**	0.005	0.002-0.011	0	0-0.002	0.00114	0.0034
**Clostridium_butyricum**	0.005	0.003-0.007	0.003	0.001-0.007	0.14373	0.43
**Alistipes_sp._5CPEGH6**	0.004	0.002-0.013	0.001	0-0.011	0.06272	0.12
**Klebsiella_pneumoniae**	0.004	0.002-0.013	0.014	0.006-0.148	0.03998	0.12
**uncultured_crAssphage**	0.004	0.003-0.007	0	0-0.004	0.00541	0.012
**Gordonibacter_pamelaeae**	0.003	0.002-0.005	0.004	0.001-0.009	1	1
**Veillonella_rodentium**	0.003	0.001-0.014	0.005	0-0.027	0.55976	1
**Salmonella_enterica**	0.003	0.002-0.004	0.021	0.01-0.081	4.20E-05	0.00013
**Ruminococcus_champanellensis**	0.003	0.002-0.006	0.002	0.001-0.009	0.17068	0.51
**Prevotella_oris**	0.003	0.001-0.007	0	0-0.002	0.00299	0.009
**Prevotella_dentalis**	0.003	0.001-0.008	0.001	0-0.003	0.02094	0.063
**Prevotella_ruminicola**	0.003	0.002-0.009	0.001	0-0.001	0.00081	0.0024
**Prevotella_melaninogenica**	0.003	0.002-0.01	0.001	0-0.002	0.0019	0.0057
**Eubacterium_callanderi**	0.003	0.001-0.005	0.003	0.001-0.014	0.98809	0.99
**Schaalia_odontolytica**	0.003	0.002-0.005	0.015	0.003-0.029	0.0102	0.031
**Streptococcus_thermophilus**	0.002	0.002-0.008	0.004	0.002-0.009	0.26013	0.78
**Bifidobacterium_animalis**	0.002	0.001-0.005	0.001	0-0.002	0.06217	0.19
**Clostridium_saccharoperbutylacetonicum**	0.002	0.001-0.004	0.002	0.001-0.004	0.34499	0.63
**Enterobacter_cloacae**	0.002	0.001-0.006	0.004	0.002-0.007	0.14373	0.43
**Bifidobacterium_dentium**	0.002	0.001-0.005	0.001	0-0.004	0.31498	0.31
**Clostridium_baratii**	0.002	0.001-0.005	0.002	0.001-0.003	0.32977	0.67
**Megasphaera_elsdenii**	0.002	0.001-0.003	0.001	0-0.003	0.10606	0.32
**Bacillus_cereus**	0.002	0.001-0.002	0.002	0.001-0.14	0.54501	0.82
**Prevotella_denticola**	0.002	0.001-0.009	0	0-0.002	0.0015	0.0045
**Prevotella_fusca**	0.002	0.001-0.006	0	0-0.001	0.00104	0.0031
**Enterococcus_avium**	0.002	0.001-0.004	0.004	0.001-0.015	0.28669	0.49
**Alistipes_sp._3BBH6**	0.001	0-0.004	0.001	0-0.003	0.16852	0.41
**Escherichia_albertii**	0.001	0.001-0.003	0.011	0.003-0.045	0.00213	0.0064
**Fusobacterium_mortiferum**	0.001	0-0.001	0	0-0.001	0.12762	0.38
**Acidaminococcus_intestini**	0.001	0.001-0.004	0.001	0-0.001	0.03162	0.095

**Megamonas_hypermegale**	0.001	0.001-0.002	0.001	0.001-0.003	0.82274	0.98
**Morganella_morganii**	0.001	0.001-0.002	0.001	0.001-0.064	0.89309	0.89
**Klebsiella_variicola**	0.001	0-0.002	0.001	0-0.004	0.27319	0.68
**Acidaminococcus_fermentans**	0.001	0.001-0.002	0.001	0-0.003	0.50079	0.5
**Phascolarctobacterium_succinatutens**	0.001	0-0.057	0	0-0	0.03544	0.11
**Chryseobacterium_taklimakanense**	0.001	0-0.001	0	0-0.001	0.01475	0.041
**Phascolarctobacterium_faecium**	0.001	0-0.045	0	0-0	0.00127	0.0038
**Actinoalloteichus_sp._AHMU_CJ021**	0	0-0	0	0-0.224	0.13404	0.2

Pre_KD, samples collected before ketogenic diet initiation.

TABLE S5 Comparison between Healthy and Pre_KD groups at KEGG pathway

KEGG Pathway	Healthy (median)	Health (Q1-Q3)	Pre_KD (median)	Pre_KD (Q1-Q3)	Healthy_vs_Pre_KD (p.value)	Healthy_vs_Pre_KD (p.adj)
Metabolic_pathways	17.217	16.917-17.373	16.548	15.945-17.094	0.0051	0.015
Biosynthesis_of_secondary_metabolites	6.788	6.694-6.889	6.837	6.745-6.868	0.50079	0.5
Biosynthesis_of_antibiotics	5.059	4.972-5.147	5.096	4.994-5.2	0.31498	0.38
Microbial_metabolism_in_diverse_environments	3.986	3.903-4.174	4.579	4.173-4.813	2.50E-05	7.50E-05
Biosynthesis_of_amino_acids	3.482	3.407-3.569	3.442	3.308-3.581	0.34499	0.34
Two_component_system	2.504	2.331-2.602	2.599	2.214-2.809	0.50079	0.98
Carbon_metabolism	2.438	2.398-2.459	2.476	2.45-2.544	0.00626	0.019
Purine_metabolism	2.087	2.026-2.105	1.967	1.923-2.011	0.00764	0.011
ABC_transporters	1.937	1.79-2.084	3.074	2.147-3.531	0.0123	0.037
Amino_sugar_and_nucleotide_sugar_metabolism	1.877	1.78-1.947	1.454	1.227-1.769	0.00055	0.0016
Ribosome	1.635	1.572-1.718	1.537	1.384-1.678	0.05008	0.075
Pyrimidine_metabolism	1.581	1.514-1.631	1.523	1.462-1.671	0.37671	0.57
Starch_and_sucrose_metabolism	1.445	1.365-1.587	1.236	1.078-1.438	0.01475	0.044
Galactose_metabolism	1.341	1.215-1.415	1.153	0.934-1.361	0.05008	0.075
Glycolysis__Gluconeogenesis	1.107	1.054-1.138	1.107	1.065-1.173	0.68663	0.69
Pyruvate_metabolism	0.96	0.916-0.98	1.013	0.953-1.059	0.01761	0.026
Alanine_aspartate_and_glutamate_metabolism	0.917	0.877-0.953	0.851	0.812-0.89	0.01613	0.048
Cysteine_and_methionine_metabolism	0.912	0.857-0.967	0.929	0.883-0.949	0.60082	0.6
Other_glycan_degradation	0.878	0.774-1.039	0.399	0.248-0.898	0.02477	0.037
Fructose_and_mannose_metabolism	0.868	0.825-0.901	0.835	0.786-0.928	0.98809	0.99
Oxidative_phosphorylation	0.852	0.811-0.901	0.829	0.73-0.868	0.11291	0.34
Homologous_recombination	0.849	0.811-0.881	0.811	0.783-0.835	0.15234	0.23
Carbon_fixation_pathways_in_prokaryotes	0.811	0.783-0.842	0.808	0.705-0.869	0.91675	0.92
Aminoacyl_tRNA_biosynthesis	0.773	0.743-0.829	0.784	0.703-0.847	0.73115	0.73
Glycine_serine_and_threonine_metabolism	0.765	0.737-0.787	0.815	0.748-0.854	0.05388	0.083
Mismatch_repair	0.723	0.672-0.788	0.684	0.644-0.725	0.09332	0.28
Pentose_phosphate_pathway	0.711	0.658-0.735	0.753	0.709-0.797	0.09332	0.14
Methane_metabolism	0.7	0.687-0.732	0.72	0.7-0.732	0.30062	0.82
xocarboxylic_acid_metabolism	0.68	0.661-0.711	0.752	0.694-0.777	0.01484	0.028
DNA_replication	0.672	0.646-0.731	0.612	0.593-0.644	0.00459	0.014
Glyoxylate_and_dicarboxylate_metabolism	0.668	0.643-0.682	0.719	0.684-0.783	0.00299	0.0069
RNA_degradation	0.635	0.619-0.658	0.562	0.514-0.581	3.00E-05	9.00E-05
beta_Lactam_resistance	0.628	0.591-0.657	0.505	0.428-0.619	0.0269	0.081
Peptidoglycan_biosynthesis	0.627	0.599-0.671	0.617	0.581-0.669	0.73115	0.73
Porphyrin_and_chlorophyll_metabolism	0.607	0.553-0.661	0.591	0.508-0.715	0.98809	0.99
Pentose_and_glucuronate_interconversions	0.602	0.568-0.651	0.588	0.514-0.617	0.26013	0.39
Phenylalanine_tyrosine_and_tryptophan_biosynthesis	0.593	0.564-0.608	0.568	0.521-0.6	0.28669	0.43
Sphingolipid_metabolism	0.587	0.511-0.735	0.284	0.137-0.63	0.02094	0.044
Citrate_cycle_TCA_cycle	0.549	0.508-0.582	0.526	0.474-0.602	0.50079	0.84

**Bacterial_secretion_system**	0.546	0.528-0.597	0.69	0.545-0.82	0.01348	0.04
**Propanoate_metabolism**	0.535	0.526-0.568	0.637	0.586-0.676	0.00092	0.0027
**Fatty_acid_metabolism**	0.502	0.491-0.529	0.486	0.448-0.513	0.12009	0.36
**Protein_export**	0.493	0.46-0.515	0.42	0.412-0.467	0.00048	0.00072
**Butanoate_metabolism**	0.489	0.457-0.523	0.596	0.526-0.69	0.0019	0.0057
**Lysine_biosynthesis**	0.483	0.454-0.496	0.475	0.446-0.494	0.50079	0.6
**One_carbon_pool_by_folate**	0.482	0.473-0.496	0.461	0.43-0.487	0.02918	0.088
**Arginine_biosynthesis**	0.482	0.461-0.488	0.467	0.446-0.486	0.52009	0.67
**Fatty_acid_biosynthesis**	0.481	0.464-0.498	0.405	0.374-0.465	0.00371	0.011
**Carbon_fixation_in_photosynthetic_organisms**	0.475	0.467-0.482	0.448	0.435-0.467	0.00333	0.01
**Glycerophospholipid_metabolism**	0.467	0.436-0.497	0.519	0.491-0.534	0.01121	0.034
**Cationic_antimicrobial_peptide_CAMP_resistance**	0.445	0.404-0.477	0.422	0.344-0.485	0.66475	0.73
**Pantothenate_and_CoA_biosynthesis**	0.426	0.415-0.442	0.465	0.422-0.501	0.08742	0.26
**Cell_cycle___Caulobacter**	0.424	0.403-0.459	0.39	0.369-0.415	0.03998	0.12
**Lysosome**	0.411	0.345-0.509	0.125	0.045-0.383	0.01475	0.044
**Streptomycin_biosynthesis**	0.406	0.369-0.453	0.396	0.365-0.476	0.91675	0.92
**Nucleotide_excision_repair**	0.4	0.368-0.429	0.337	0.311-0.358	3.00E-05	9.00E-05
**Thiamine_metabolism**	0.394	0.371-0.412	0.352	0.314-0.39	0.01921	0.058
**Nicotinate_and_nicotinamide_metabolism**	0.393	0.379-0.41	0.427	0.402-0.448	0.0579	0.17
**Folate_biosynthesis**	0.393	0.36-0.418	0.416	0.365-0.433	0.37671	0.43
**Nitrogen_metabolism**	0.382	0.358-0.404	0.391	0.365-0.476	0.27319	0.67
**Base_excision_repair**	0.379	0.364-0.411	0.346	0.335-0.358	8.20E-05	0.00025
**Biotin_metabolism**	0.366	0.335-0.4	0.32	0.281-0.36	0.03423	0.1
**Arginine_and_proline_metabolism**	0.353	0.343-0.374	0.388	0.358-0.432	0.0269	0.081
**Histidine_metabolism**	0.349	0.338-0.368	0.346	0.31-0.378	0.96427	0.96
**Glycosaminoglycan_degradation**	0.34	0.261-0.42	0.101	0.061-0.301	0.0123	0.037
**Valine_leucine_and_isoleucine_biosynthesis**	0.339	0.322-0.372	0.422	0.342-0.475	0.00692	0.021
**Cyanoamino_acid_metabolism**	0.333	0.279-0.416	0.228	0.186-0.318	0.00213	0.0064
**Glycerolipid_metabolism**	0.329	0.291-0.347	0.339	0.319-0.35	0.31498	0.31
**Terpenoid_backbone_biosynthesis**	0.325	0.315-0.348	0.322	0.312-0.344	0.41013	0.51
**Selenocompound_metabolism**	0.324	0.31-0.345	0.376	0.338-0.394	0.0015	0.0045
**Lipopolysaccharide_biosynthesis**	0.322	0.274-0.357	0.341	0.221-0.41	0.75375	0.78
**Bacterial_chemotaxis**	0.319	0.267-0.381	0.312	0.266-0.34	0.55976	0.78
**other**	0.308	0.253-0.345	0.418	0.317-0.461	0.0015	0.0032
**Sulfur_metabolism**	0.306	0.294-0.333	0.377	0.307-0.434	0.01475	0.044
**Phosphotransferase_system_PTS**	0.305	0.262-0.554	0.788	0.42-0.971	0.00267	0.008
**Longevity_regulating_pathway___multiple_species**	0.275	0.24-0.334	0.121	0.1-0.282	0.00413	0.012
**Glycosphingolipid_biosynthesis___globo_series**	0.274	0.221-0.341	0.099	0.058-0.262	0.01475	0.044
**Drug_metabolism___other_enzymes**	0.266	0.257-0.278	0.247	0.228-0.276	0.11291	0.34
**Vancomycin_resistance**	0.263	0.234-0.279	0.239	0.216-0.268	0.14373	0.34
**Phenylpropanoid_biosynthesis**	0.254	0.212-0.345	0.184	0.128-0.233	0.0019	0.0057
**Photosynthesis**	0.248	0.229-0.288	0.247	0.211-0.284	0.64314	0.64

**Endocytosis**	0.234	0.159-0.31	0.299	0.141-0.401	0.53975	0.81
**Valine_leucine_and_isoleucine_degradation**	0.233	0.22-0.244	0.252	0.222-0.276	0.07145	0.11
**Sulfur_relay_system**	0.232	0.224-0.246	0.277	0.237-0.315	0.02094	0.063
**Monobactam_biosynthesis**	0.221	0.208-0.226	0.218	0.205-0.223	0.79957	0.8
**Polyketide_sugar_unit_biosynthesis**	0.22	0.202-0.255	0.236	0.203-0.273	0.68663	0.69
**Phenylalanine_metabolism**	0.207	0.199-0.221	0.252	0.205-0.332	0.0269	0.081
**Meiosis___yeast**	0.202	0.179-0.261	0.05	0.011-0.172	0.00092	0.0027
**C5_Branched_dibasic_acid_metabolism**	0.202	0.192-0.219	0.246	0.22-0.301	0.00024	0.00071
**Riboflavin_metabolism**	0.187	0.177-0.206	0.164	0.145-0.193	0.13549	0.2
**Glutathione_metabolism**	0.186	0.171-0.198	0.222	0.189-0.25	0.02094	0.059
**Ubiquinone_and_other_terpenoid_quinone_biosynthesis**	0.186	0.171-0.206	0.228	0.17-0.271	0.07145	0.21
**Fatty_acid_degradation**	0.184	0.17-0.206	0.241	0.19-0.289	0.00169	0.0051
**Central_carbon_metabolism_in_cancer**	0.181	0.169-0.192	0.168	0.137-0.185	0.11291	0.22
**Vitamin_B6_metabolism**	0.179	0.172-0.199	0.192	0.179-0.205	0.4452	0.45
**Protein_digestion_and_absorption**	0.177	0.144-0.237	0.209	0.166-0.255	0.46334	0.87
**Amoebiasis**	0.175	0.11-0.21	0.199	0.136-0.293	0.24749	0.37
**Peroxisome**	0.173	0.164-0.179	0.177	0.168-0.197	0.34499	0.87
**Tyrosine_metabolism**	0.17	0.158-0.182	0.202	0.173-0.276	0.00626	0.019
**Glycosphingolipid_biosynthesis___ganglio_series**	0.165	0.136-0.207	0.035	0.009-0.153	0.01475	0.044
**Inositol_phosphate_metabolism**	0.164	0.141-0.199	0.155	0.118-0.177	0.2121	0.25
**Longevity_regulating_pathway___worm**	0.151	0.141-0.168	0.158	0.149-0.176	0.22348	0.59
**RNA_polymerase**	0.144	0.134-0.156	0.147	0.136-0.163	0.42746	0.69
**RNA_transport**	0.141	0.131-0.167	0.136	0.105-0.157	0.31498	0.33
**Degradation_of_aromatic_compounds**	0.139	0.122-0.167	0.254	0.157-0.294	0.00024	0.00071
**Tuberculosis**	0.129	0.115-0.139	0.119	0.113-0.145	0.55976	0.56
**Glucagon_signaling_pathway**	0.128	0.12-0.14	0.135	0.113-0.159	0.70877	0.96
**PPAR_signaling_pathway**	0.127	0.122-0.134	0.112	0.074-0.127	0.02279	0.068
**Biosynthesis_of_unsaturated_fatty_acids**	0.123	0.111-0.128	0.129	0.117-0.134	0.17068	0.47
**Acarbose_and_validamycin_biosynthesis**	0.123	0.113-0.141	0.147	0.116-0.17	0.36064	0.54
**GABAergic_synapse**	0.116	0.106-0.126	0.098	0.09-0.113	0.00692	0.021
**Plant_pathogen_interaction**	0.116	0.1-0.121	0.091	0.08-0.106	0.01121	0.034
**Ascorbate_and_aldarate_metabolism**	0.115	0.098-0.125	0.157	0.119-0.219	0.00842	0.013
**D_Glutamine_and_D_glutamate_metabolism**	0.114	0.109-0.118	0.109	0.103-0.117	0.22348	0.34
**Legionellosis**	0.112	0.11-0.117	0.111	0.101-0.122	0.77657	0.78
**Novobiocin_biosynthesis**	0.11	0.103-0.115	0.108	0.1-0.115	0.79957	0.8
**Benzoate_degradation**	0.11	0.096-0.124	0.172	0.125-0.251	0.00013	0.00039
**Vibrio_cholerae_infection**	0.107	0.07-0.157	0.135	0.083-0.256	0.15234	0.46
**beta_Alanine_metabolism**	0.105	0.098-0.124	0.125	0.101-0.203	0.09332	0.14
**Taurine_and_hypotaurine_metabolism**	0.105	0.096-0.109	0.109	0.101-0.126	0.08742	0.26
**MicroRNAs_in_cancer**	0.104	0.088-0.122	0.079	0.062-0.096	0.05388	0.16
**Pertussis**	0.102	0.084-0.116	0.14	0.08-0.162	0.15234	0.46
**Phosphonate_and_phosphinate_metabolism**	0.099	0.088-0.115	0.119	0.109-0.154	0.07649	0.16

**Glutamatergic_synapse**	0.095	0.09-0.101	0.076	0.067-0.089	0.00042	0.0012
**Insulin_resistance**	0.095	0.085-0.107	0.088	0.075-0.113	0.60082	0.7
**Tropane_piperidine_and_pyridine_alkaloid_biosynthesis**	0.093	0.088-0.099	0.104	0.087-0.112	0.13549	0.4
**Lysine_degradation**	0.092	0.08-0.108	0.133	0.099-0.204	0.00299	0.009
**Aminobenzoate_degradation**	0.092	0.081-0.101	0.12	0.103-0.129	0.00092	0.0027
**Adipocytokine_signaling_pathway**	0.092	0.082-0.095	0.081	0.048-0.096	0.22348	0.67
**Butirosin_and_neomycin_biosynthesis**	0.089	0.063-0.099	0.085	0.069-0.095	0.84607	0.85
**Protein_processing_in_endoplasmic_reticulum**	0.089	0.084-0.102	0.075	0.042-0.09	0.01348	0.02
**Flagellar_assembly**	0.088	0.052-0.124	0.245	0.105-0.303	0.00213	0.0064
**Salmonella_infection**	0.088	0.069-0.106	0.107	0.092-0.15	0.09332	0.22
**AMPK_signaling_pathway**	0.079	0.069-0.09	0.061	0.042-0.079	0.0579	0.17
**HIF_1_signaling_pathway**	0.078	0.074-0.084	0.074	0.071-0.086	0.50079	0.78
**D_Alanine_metabolism**	0.075	0.068-0.082	0.08	0.073-0.082	0.37671	0.46
**Biosynthesis_of_ansamycins**	0.074	0.064-0.083	0.073	0.063-0.082	0.86952	0.87
**Insulin_signaling_pathway**	0.074	0.067-0.08	0.072	0.068-0.077	0.68663	0.98
**Chloroalkane_and_chloroalkene_degradation**	0.073	0.055-0.087	0.118	0.074-0.131	0.0102	0.031
**Phosphatidylinositol_signaling_system**	0.073	0.068-0.078	0.071	0.066-0.081	0.86952	0.87
**N_Glycan_biosynthesis**	0.07	0.058-0.08	0.044	0.025-0.057	0.0019	0.0057
**Tetracycline_biosynthesis**	0.066	0.056-0.075	0.085	0.069-0.094	0.0123	0.037
**mRNA_surveillance_pathway**	0.063	0.053-0.083	0.074	0.049-0.095	0.66475	0.66
**Steroid_hormone_biosynthesis**	0.063	0.05-0.098	0.022	0.011-0.081	0.03423	0.051
**Epithelial_cell_signaling_in_Helicobacter_pylori_infection**	0.062	0.06-0.067	0.066	0.06-0.073	0.27319	0.41
**Vibrio_cholerae_pathogenic_cycle**	0.06	0.055-0.067	0.068	0.054-0.091	0.31498	0.47
**Choline_metabolism_in_cancer**	0.059	0.036-0.079	0.051	0.044-0.071	0.73115	0.73
**Carbapenem_biosynthesis**	0.058	0.056-0.063	0.058	0.051-0.063	0.70877	0.71
**Secondary_bile_acid_biosynthesis**	0.057	0.051-0.062	0.037	0.02-0.054	0.00299	0.009
**Tryptophan_metabolism**	0.057	0.042-0.064	0.101	0.066-0.14	0.00015	0.00023
**Bacterial_invasion_of_epithelial_cells**	0.056	0.043-0.09	0.069	0.058-0.081	0.32977	0.33
**Pathways_in_cancer**	0.055	0.047-0.06	0.046	0.036-0.06	0.2121	0.32
**Primary_bile_acid_biosynthesis**	0.054	0.045-0.059	0.03	0.016-0.053	0.00842	0.025
**PI3K_Akt_signaling_pathway**	0.054	0.05-0.061	0.047	0.035-0.059	0.19059	0.21
**Naphthalene_degradation**	0.053	0.033-0.06	0.081	0.055-0.105	0.00565	0.017
**Isoquinoline_alkaloid_biosynthesis**	0.051	0.047-0.053	0.053	0.05-0.059	0.2121	0.21
**Penicillin_and_cephalosporin_biosynthesis**	0.05	0.04-0.063	0.032	0.025-0.052	0.04314	0.13
**Biosynthesis_of_vancomycin_group_antibiotics**	0.05	0.043-0.055	0.046	0.04-0.055	0.52009	0.52
**Metabolism_of_xenobiotics_by_cytochrome_P450**	0.049	0.033-0.059	0.076	0.046-0.094	0.01613	0.048
**Type_I_diabetes_mellitus**	0.048	0.045-0.051	0.052	0.047-0.06	0.10606	0.32
**Pathogenic_Escherichia_coli_infection**	0.046	0.03-0.071	0.042	0.024-0.063	0.50079	0.54
**NOD_like_receptor_signaling_pathway**	0.044	0.039-0.06	0.028	0.018-0.041	0.00118	0.0018
**Zeatin_biosynthesis**	0.043	0.039-0.046	0.03	0.027-0.041	0.0051	0.015
**Progesterone_mediated_oocyte_maturation**	0.042	0.038-0.051	0.036	0.029-0.041	0.0269	0.081

**Lipoic_acid_metabolism**	0.041	0.031-0.047	0.038	0.034-0.046	0.75375	0.98
**Nitrotoluene_degradation**	0.041	0.027-0.054	0.063	0.048-0.094	0.00239	0.0036
**Retinol_metabolism**	0.041	0.035-0.051	0.055	0.041-0.059	0.12762	0.19
**Proteoglycans_in_cancer**	0.039	0.036-0.044	0.032	0.029-0.037	0.01613	0.024
**Alzheimers_disease**	0.039	0.036-0.043	0.04	0.037-0.048	0.41013	0.71
**Huntingtons_disease**	0.039	0.034-0.052	0.069	0.057-0.107	0.00081	0.002
**Ribosome_biogenesis_in_eukaryotes**	0.038	0.034-0.045	0.042	0.04-0.047	0.12762	0.15
**Type_II_diabetes_mellitus**	0.038	0.035-0.041	0.037	0.033-0.042	0.58013	0.96
**Antigen_processing_and_presentation**	0.037	0.033-0.044	0.026	0.016-0.034	0.00118	0.0018
**Regulation_of_actin_cytoskeleton**	0.037	0.021-0.067	0.036	0.023-0.058	0.62183	0.71
**Viral_carcinogenesis**	0.036	0.032-0.044	0.032	0.031-0.037	0.09953	0.3
**Apoptosis___fly**	0.035	0.03-0.04	0.031	0.026-0.043	0.73115	0.73
**Primary_immunodeficiency**	0.034	0.032-0.04	0.035	0.031-0.037	0.62183	0.89
**Estrogen_signaling_pathway**	0.033	0.029-0.038	0.023	0.016-0.031	0.00213	0.0064
**Prostate_cancer**	0.033	0.029-0.038	0.023	0.016-0.031	0.00224	0.0067
**Adherens_junction**	0.031	0.015-0.063	0.032	0.021-0.052	0.96427	0.96
**Apoptosis**	0.03	0.019-0.037	0.006	0.002-0.024	0.00055	0.0016
**Spliceosome**	0.03	0.019-0.037	0.038	0.024-0.053	0.15234	0.46
**AGE_RAGE_signaling_pathway_in_diabetic_complications**	0.03	0.024-0.037	0.036	0.022-0.054	0.42746	0.43
**Carbohydrate_digestion_and_absorption**	0.029	0.016-0.037	0.025	0.02-0.036	0.70877	0.96
**Fc_gamma_R_mediated_phagocytosis**	0.028	0.018-0.058	0.044	0.029-0.048	0.52009	0.78
**Shigellosis**	0.026	0.013-0.056	0.039	0.021-0.048	0.66475	0.66
**FoxO_signaling_pathway**	0.026	0.02-0.032	0.035	0.028-0.039	0.04651	0.07
**Drug_metabolism___cytochrome_P450**	0.026	0.017-0.036	0.057	0.035-0.076	0.00413	0.012
**Chemokine_signaling_pathway**	0.025	0.013-0.053	0.032	0.02-0.043	0.86952	0.87
**Biosynthesis_of_siderophore_group_nonribosomal_peptides**	0.025	0.018-0.032	0.057	0.036-0.091	0.00104	0.0031
**Longevity_regulating_pathway**	0.024	0.02-0.029	0.031	0.026-0.039	0.08742	0.13
**Thyroid_hormone_synthesis**	0.024	0.018-0.028	0.023	0.018-0.028	0.77657	0.87
**Focal_adhesion**	0.023	0.02-0.03	0.025	0.013-0.038	0.77657	0.78
**Proximal_tubule_bicarbonate_reclamation**	0.023	0.019-0.03	0.024	0.017-0.03	0.62183	0.73
**Platinum_drug_resistance**	0.022	0.015-0.029	0.047	0.022-0.064	0.0123	0.037
**Stilbenoid_diarylheptanoid_and_gingerol_biosynthesis**	0.022	0.018-0.024	0.013	0.005-0.022	0.0269	0.081
**Flavonoid_biosynthesis**	0.022	0.018-0.024	0.013	0.005-0.022	0.0269	0.081
**MAPK_signaling_pathway___yeast**	0.022	0.016-0.036	0.025	0.022-0.031	0.37671	0.84
**Platelet_activation**	0.021	0.016-0.027	0.027	0.018-0.037	0.37671	0.38
**Dorso_ventral_axis_formation**	0.021	0.009-0.033	0.031	0.019-0.049	0.15234	0.23
**Rheumatoid_arthritis**	0.019	0.013-0.022	0.003	0-0.014	0.00141	0.0025
**ECM_receptor_interaction**	0.019	0.016-0.024	0.028	0.013-0.036	0.30062	0.45
**Herpes_simplex_infection**	0.019	0.014-0.029	0.023	0.018-0.043	0.26013	0.49
**Flavone_and_flavonol_biosynthesis**	0.019	0.009-0.024	0.015	0.009-0.022	0.52009	0.95

**Epstein_Barr_virus_infection**	0.018	0.013-0.03	0.022	0.018-0.043	0.32977	0.7
**Arachidonic_acid_metabolism**	0.018	0.013-0.023	0.02	0.012-0.026	0.77657	0.78
**MAPK_signaling_pathway___fly**	0.017	0.015-0.019	0.021	0.018-0.024	0.07145	0.11
**Salivary_secretion**	0.016	0.012-0.025	0.014	0.007-0.052	0.77657	0.78
**Phospholipase_D_signaling_pathway**	0.016	0.013-0.019	0.014	0.01-0.016	0.22348	0.67
**Osteoclast_differentiation**	0.015	0.013-0.018	0.001	0-0.012	0.00156	0.0047
**Amyotrophic_lateral_sclerosis_ALS**	0.015	0.009-0.021	0.029	0.018-0.041	0.00371	0.0062
**Plant_hormone_signal_transduction**	0.015	0.01-0.02	0.018	0.002-0.027	0.82274	0.82
**Limonene_and_pinene_degradation**	0.015	0.013-0.02	0.038	0.019-0.057	0.00299	0.009
**Prolactin_signaling_pathway**	0.015	0.009-0.02	0.02	0.012-0.03	0.15234	0.23
**Caprolactam_degradation**	0.014	0.01-0.016	0.018	0.009-0.031	0.27319	0.41
**Biosynthesis_of_type_II_polyketide_products**	0.013	0.009-0.018	0.014	0.008-0.018	0.77657	0.78
**GlycosylphosphatidylinositolGPI_anchor_biosynthesis**	0.013	0.008-0.017	0.004	0.001-0.022	0.42746	0.64
**Nonribosomal_peptide_structures**	0.012	0.009-0.021	0.017	0.013-0.021	0.27319	0.59
**Synthesis_and_degradation_of_ketone_bodies**	0.012	0.009-0.014	0.03	0.011-0.041	0.03162	0.081
**Glycosphingolipid_biosynthesis___lacto_and_neolacto_series**	0.012	0.007-0.021	0	0-0.004	0.00114	0.0034
**Staphylococcus_aureus_infection**	0.011	0.007-0.014	0.007	0.006-0.013	0.37671	0.6
**Cell_cycle___yeast**	0.01	0.007-0.015	0.013	0.01-0.015	0.42746	0.87
**Ubiquitin_mediated_proteolysis**	0.01	0.007-0.014	0.012	0.006-0.015	0.98809	0.99
**Cell_cycle**	0.009	0.007-0.014	0.012	0.006-0.015	0.89309	0.99
**Styrene_degradation**	0.009	0.006-0.016	0.017	0.012-0.028	0.00842	0.025
**Small_cell_lung_cancer**	0.009	0.006-0.014	0.006	0.003-0.019	0.60082	0.67
**Chlorocyclohexane_and_chlorobenzene_degradation**	0.008	0.005-0.012	0.024	0.013-0.03	2.10E-05	6.30E-05
**Calcium_signaling_pathway**	0.008	0.004-0.011	0.009	0.006-0.01	0.46334	0.46
**Xylene_degradation**	0.008	0.006-0.012	0.023	0.015-0.043	0.00015	0.00046
**Ether_lipid_metabolism**	0.007	0.004-0.013	0.018	0.012-0.024	0.00133	0.004
**Dioxin_degradation**	0.007	0.004-0.01	0.023	0.012-0.034	0.00081	0.0024
**Arrhythmogenic_right_ventricular_cardiomyopathy_ARVC**	0.004	0.001-0.01	0.002	0-0.008	0.24749	0.56
**Leukocyte_transendothelial_migration**	0.004	0-0.011	0	0-0.002	0.01479	0.044
**Geraniol_degradation**	0.003	0.003-0.005	0.024	0.007-0.039	0.00013	0.00039
**Tight_junction**	0.002	0.001-0.008	0.001	0-0.006	0.31067	0.73
**Chemical_carcinogenesis**	0.001	0.001-0.004	0.019	0.007-0.041	4.20E-05	0.00013
**Systemic_lupus_erythematosus**	0	0-0.003	0	0-0	0.01142	0.034

Pre_KD, samples collected before ketogenic diet initiation.

TABLE S6 Paired comparison between Pre_KD and Post_KD groups at genus level

Genus	Pre_KD (median)	Pre_KD (Q1-Q3)	Post_KD (median)	Post_KD (Q1-Q3)	Pre_KD_vs_Post_KD (p.value)	Pre_KD_vs_Post_KD (p.adj)
Unclassified	41.696	29.457-55.012	42.426	22.731-54.825	0.9697	0.97
Escherichia	7.566	0.957-10.726	2.518	0.739-4.121	0.2036	0.2
Bifidobacterium	6.082	2.117-10.979	1.244	0.227-2.623	0.0049	0.0049
Bacteroides	3.295	0.189-43.282	21.179	0.834-44.56	0.3804	0.38
Flavonifractor	1.271	0.256-1.619	0.508	0.255-1.689	0.6221	0.62
Veillonella	1.048	0.013-3.262	0.251	0.045-1.275	0.3394	0.34
Lachnoclostridium	0.853	0.563-1.483	0.587	0.365-2.962	0.4238	0.42
other	0.807	0.365-1.126	0.909	0.408-1.39	1	1
Blautia	0.325	0.117-0.753	0.356	0.206-1.386	0.1294	0.13
Streptococcus	0.152	0.085-0.332	0.12	0.067-0.187	0.0923	0.092
Parabacteroides	0.143	0.024-0.396	0.349	0.048-2.136	0.0342	0.034
Faecalibacterium	0.128	0.014-1.229	0.18	0.035-0.453	0.9697	0.97
Clostridioides	0.124	0.072-0.174	0.139	0.081-0.246	0.6772	0.68
Roseburia	0.092	0.021-0.275	0.101	0.043-0.208	0.6772	0.68
Clostridium	0.09	0.032-0.13	0.089	0.04-0.24	0.2661	0.27
Ruthenibacterium	0.075	0.013-0.18	0.067	0.014-0.165	0.7334	0.73
Citrobacter	0.073	0.016-0.142	0.028	0.012-0.053	0.0522	0.052
Klebsiella	0.067	0.025-0.571	0.025	0.008-0.11	0.4238	0.42
Enterococcus	0.066	0.028-0.11	0.037	0.019-0.081	0.1294	0.13
Eggerthella	0.063	0.031-0.237	0.138	0.034-0.324	0.3013	0.3
Eubacterium	0.06	0.015-0.321	0.074	0.041-0.166	0.9097	0.91
Bacillus	0.054	0.024-0.225	0.087	0.023-0.143	0.5693	0.57
Dysosmobacter	0.05	0.013-0.09	0.066	0.025-0.204	0.5186	0.52
Alistipes	0.047	0.004-0.24	0.096	0.004-0.562	0.1763	0.18
Anaerostipes	0.047	0.016-0.422	0.341	0.12-0.707	0.1099	0.11
Pseudomonas	0.04	0.014-0.048	0.04	0.013-0.047	0.791	0.79
Hungatella	0.038	0.003-0.052	0.026	0.016-0.075	0.5186	0.52
Paenibacillus	0.037	0.008-0.051	0.036	0.023-0.071	0.3013	0.3
Anaerobutyricum	0.031	0.012-0.077	0.045	0.025-0.069	0.7334	0.73
Salmonella（沙门）	0.025	0.015-0.093	0.009	0.004-0.023	0.0342	0.034
Lactobacillus	0.025	0.015-0.032	0.021	0.012-0.036	0.8501	0.85
Mordavella	0.024	0.004-0.043	0.022	0.011-0.043	0.791	0.79
Shigella（志贺）	0.024	0.014-0.112	0.009	0.004-0.022	0.0068	0.0068
Enterobacter	0.022	0.011-0.058	0.009	0.006-0.048	0.4697	0.47
Actinomyces	0.019	0.005-0.036	0.006	0.003-0.02	0.1099	0.11
Intestinimonas	0.017	0.007-0.043	0.029	0.007-0.066	1	1
Christensenella	0.017	0.005-0.036	0.02	0.011-0.03	0.8501	0.85
Oscillibacter	0.016	0.007-0.041	0.025	0.009-0.047	0.7334	0.73
Streptomyces	0.016	0.004-0.026	0.016	0.008-0.03	0.9697	0.97

Longibaculum	0.015	0.003-0.026	0.011	0.007-0.024	0.791	0.79
Schaalia	0.014	0.004-0.028	0.006	0.002-0.017	0.4238	0.42
Odoribacter	0.013	0-0.028	0.026	0.007-0.227	0.021	0.021
Ruminococcus	0.013	0.004-0.116	0.007	0.006-0.045	0.9097	0.91
Staphylococcus	0.012	0.009-0.017	0.01	0.006-0.022	0.7334	0.73
Prevotella	0.012	0.006-0.022	0.027	0.022-0.036	0.3394	0.34
Campylobacter	0.011	0.005-0.023	0.024	0.008-0.036	0.1514	0.15
Fusobacterium	0.011	0.007-0.016	0.007	0.005-0.034	0.2334	0.23
Corynebacterium	0.01	0.005-0.019	0.007	0.002-0.012	0.1763	0.18
Butyrivibrio	0.01	0.004-0.023	0.012	0.005-0.029	0.8501	0.85
Faecalitalea	0.01	0.005-0.027	0.015	0.007-0.032	0.9697	0.97
Paraprevotella	0.01	0.001-0.039	0.024	0.004-0.116	0.2334	0.23
Chryseobacterium	0.009	0.005-0.014	0.017	0.009-0.019	0.2334	0.23
Haemophilus	0.008	0-0.065	0.015	0.002-0.069	0.8501	0.85
Butyricimonas	0.007	0.001-0.019	0.011	0.004-0.099	0.3013	0.3
Desulfovibrio	0.007	0.001-0.059	0.019	0.013-0.042	0.9097	0.91
Lactococcus	0.006	0.004-0.043	0.01	0.005-0.022	0.5186	0.52
Rothia	0.006	0.004-0.025	0.002	0.001-0.007	0.0269	0.027
Megasphaera	0.005	0.001-0.011	0.005	0.003-0.008	0.791	0.79
Monoglobus	0.005	0.003-0.009	0.008	0.002-0.013	0.6221	0.62
Akkermansia	0.004	0.002-0.01	0.008	0.003-0.089	0.5693	0.57
Flavobacterium	0.004	0.003-0.007	0.008	0.005-0.015	0.1099	0.11
Gordonibacter	0.004	0.003-0.028	0.01	0.003-0.014	0.9097	0.91
Collinsella	0.004	0-0.039	0.003	0.001-0.085	0.9697	0.97
Muribaculum	0.003	0.001-0.006	0.012	0.002-0.028	0.064	0.064
Barnesiella	0.002	0.001-0.006	0.012	0.003-0.022	0.0024	0.0024
Acidaminococcus	0.002	0.001-0.008	0.003	0.002-0.01	0.791	0.79
Phascolarctobacterium	0.002	0.001-0.003	0.002	0.001-0.057	1	1
Sutterella	0.001	0-0.002	0	0-0.002	0.5693	0.57
Morganella	0.001	0.001-0.053	0.002	0.001-0.054	0.4238	0.42
Tannerella	0.001	0-0.003	0.004	0.001-0.008	0.0923	0.092
Porphyromonas	0.001	0.001-0.011	0.006	0.001-0.014	0.5186	0.52
Duncaniella	0.001	0.001-0.003	0.006	0-0.013	0.3804	0.38
Megamonas	0.001	0.001-0.003	0.001	0.001-0.002	0.6221	0.62
Actinoalloteichus	0.001	0-0.192	0.001	0-0.091	0.2036	0.2

Pre_KD, samples collected before ketogenic diet initiation; Post_KD, samples collected after one week of ketogenic diet therapy.

TABLE S7 Paired comparison between Pre_KD and Post_KD groups at species level

Species	Pre_KD (median)	Pre_KD (Q1-Q3)	Post_KD (median)	Post_KD (Q1-Q3)	Pre_KD_vs_Post_KD (p.value)	Pre_KD_vs_Post_KD (p.adj)
Unclassified	44.929	32.39-59.257	52.197	34.928-58.839	0.6772	0.68
Escherichia_coli	7.003	0.889-9.972	2.317	0.689-3.811	0.2036	0.2
other	1.741	1.375-2.138	1.621	0.643-2.542	0.8501	0.85
Bifidobacterium_breve	1.499	0.451-4.496	0.171	0.051-0.553	0.0122	0.012
Flavonifractor_plautii	1.271	0.256-1.619	0.508	0.255-1.689	0.6221	0.62
Veillonella_parvula	0.691	0.009-3.051	0.14	0.014-0.83	0.5693	0.57
X.Clostridium._bolteae	0.414	0.184-1.083	0.186	0.09-1.723	0.4697	0.47
Bacteroides_fragilis	0.312	0.025-6.087	2.743	0.126-5.584	0.5693	0.57
Bifidobacterium_longum	0.18	0.089-2.405	0.066	0.018-0.166	0.0034	0.0034
Faecalibacterium_prausnitzii	0.128	0.014-1.229	0.18	0.035-0.453	0.9697	0.97
Lachnoclostridium_sp._YL32	0.125	0.034-0.236	0.107	0.051-0.29	0.2036	0.2
Clostridioides_difficile	0.124	0.072-0.174	0.139	0.081-0.246	0.6772	0.68
Bacteroides_uniformis	0.101	0.005-0.327	0.217	0.08-1.85	0.2036	0.2
Veillonella_dispar	0.083	0.003-0.225	0.022	0.005-0.194	0.4238	0.42
Bacteroides_thetaiotaomicron	0.077	0.015-4.45	0.259	0.031-1.868	0.6772	0.68
Parabacteroides_distasonis	0.077	0.015-0.163	0.192	0.022-1.52	0.0269	0.027
Ruthenibacterium_lactatiformans	0.075	0.013-0.18	0.067	0.014-0.165	0.7334	0.73
Lachnospiraceae_bacterium_Choco86	0.071	0.024-0.191	0.096	0.05-0.303	0.2036	0.2
Eggerthella_lenta	0.062	0.03-0.234	0.136	0.033-0.321	0.3013	0.3
Lachnospiraceae_bacterium_GAM79	0.055	0.01-0.146	0.05	0.036-0.168	0.8501	0.85
Blautia_producta	0.054	0.011-0.087	0.065	0.018-0.122	0.0771	0.077
Blautia_sp._N6H1_15	0.052	0.015-0.102	0.061	0.038-0.138	0.1763	0.18
Dysosmobacter_welbionis	0.05	0.013-0.09	0.066	0.025-0.204	0.5186	0.52
Roseburia_intestinalis	0.049	0.01-0.1	0.048	0.022-0.109	0.6772	0.68
Blautia_sp._SC05B48	0.048	0.016-0.18	0.093	0.034-0.158	0.791	0.79
Blautia_hansenii	0.048	0.013-0.104	0.043	0.032-0.17	0.1294	0.13
Clostridiales_bacterium_CCNA10	0.048	0.026-0.136	0.075	0.023-0.143	0.6221	0.62
Bacteroides_xylanisolvens	0.046	0.006-0.561	0.229	0.008-0.641	0.6221	0.62
Bacteroides_vulgatus	0.045	0.003-2.428	0.219	0.034-2.441	0.6221	0.62
X.Clostridium._scindens	0.045	0.009-0.069	0.068	0.025-0.126	0.3804	0.38
Hungatella_hathewayi	0.038	0.003-0.052	0.026	0.016-0.075	0.5186	0.52
Lachnoclostridium_phocaeense	0.036	0.006-0.049	0.026	0.017-0.047	0.7334	0.73
Roseburia_hominis	0.035	0.01-0.154	0.052	0.016-0.1	0.6772	0.68
Bacteroides_ovatus	0.033	0.01-0.329	0.233	0.013-0.672	0.8501	0.85
Anaerobutyricum_hallii	0.031	0.012-0.077	0.045	0.025-0.069	0.7334	0.73
Lachnospiraceae_bacterium_KGMB03038	0.027	0.005-0.041	0.023	0.012-0.05	0.5693	0.57
X.Clostridium._sphenoides	0.027	0.003-0.035	0.014	0.011-0.018	1	1
X.Clostridium._saccharolyticum	0.026	0.003-0.036	0.014	0.01-0.022	0.9097	0.91
Bacteroides_sp._A1C1	0.025	0.003-0.19	0.069	0.021-0.369	0.1763	0.18

**Bifidobacterium_bifidum**	0.025	0.002-0.06	0.005	0.001-0.051	0.1763	0.18
**Mordavella_sp._Marseille_P3756**	0.024	0.004-0.043	0.022	0.011-0.043	0.791	0.79
**Bacteroides_caccae**	0.023	0.003-0.12	0.08	0.006-0.266	0.9097	0.91
**X.Eubacterium._rectale**	0.023	0.005-0.075	0.025	0.011-0.124	0.6772	0.68
**Anaerostipes_hadrus**	0.022	0.006-0.137	0.061	0.015-0.304	1	1
**Salmonella_enterica**	0.021	0.01-0.085	0.007	0.003-0.02	0.0425	0.042
**Bacteroides_dorei**	0.019	0.001-0.095	0.047	0.01-0.348	1	1
**Enterococcus_faecium**	0.019	0.013-0.028	0.02	0.01-0.03	0.6221	0.62
**Anaerostipes_rhamnosivorans**	0.018	0.01-0.027	0.034	0.016-0.259	0.0093	0.0093
**Intestinimonas_butyriciproducens**	0.017	0.007-0.043	0.029	0.007-0.066	1	1
**Veillonella_rodentium**	0.016	0.001-0.028	0.007	0.003-0.02	0.4238	0.42
**Longibaculum_sp._KGMB06250**	0.015	0.003-0.026	0.011	0.007-0.024	0.791	0.79
**Bacteroides_caecimuris**	0.015	0.004-0.105	0.104	0.007-0.181	0.6772	0.68
**Klebsiella_pneumoniae**	0.014	0.006-0.249	0.005	0.002-0.031	0.2661	0.27
**Bacteroides_salanitronis**	0.013	0.007-0.032	0.022	0.007-0.122	0.3394	0.34
**Odoribacter_splanchnicus**	0.013	0-0.028	0.026	0.007-0.227	0.021	0.021
**Bifidobacterium_pseudocatenulatum**	0.012	0.002-0.674	0.016	0.005-0.389	0.6772	0.68
**Schaalia_odontolytica**	0.012	0.003-0.026	0.005	0.001-0.013	0.3804	0.38
**Bacteroides_cellulosilyticus**	0.011	0-0.027	0.02	0.012-0.051	0.2036	0.2
**Escherichia_albertii**	0.011	0.005-0.042	0.004	0.002-0.017	0.0425	0.042
**Oscillibacter_sp._PEA192**	0.011	0.004-0.031	0.015	0.008-0.039	0.5186	0.52
**Streptococcus_parasanguinis**	0.011	0.003-0.028	0.002	0.001-0.005	0.0771	0.077
**Bacteroides_intestinalis**	0.011	0.001-0.031	0.026	0.011-0.098	0.0923	0.092
**Clostridium_sp._SY8519**	0.01	0.002-0.014	0.007	0.004-0.019	0.3394	0.34
**Paraprevotella_xylaniphila**	0.01	0.001-0.039	0.024	0.004-0.116	0.2334	0.23
**Faecalitalea_cylindroides**	0.01	0.005-0.027	0.015	0.007-0.032	0.9697	0.97
**X.Eubacterium._eligens**	0.01	0.002-0.049	0.011	0.004-0.029	0.6772	0.68
**Eubacterium_limosum**	0.01	0.003-0.045	0.016	0.011-0.031	0.9697	0.97
**Streptococcus_mitis**	0.009	0.004-0.012	0.003	0.001-0.006	0.0923	0.092
**Streptococcus_salivarius**	0.009	0.002-0.031	0.001	0-0.002	0.2661	0.27
**Erysipelotrichaceae_bacterium_GAM147**	0.009	0.004-0.012	0.014	0.008-0.024	0.0122	0.012
**Parabacteroides_sp._CT06**	0.008	0.001-0.029	0.024	0.001-0.063	0.0923	0.092
**Christensenella_minuta**	0.007	0.003-0.017	0.008	0.005-0.02	0.8501	0.85
**Streptococcus_sp._HSISM1**	0.007	0.002-0.024	0.001	0-0.003	0.064	0.064
**Butyricimonas_faecalis**	0.007	0.001-0.019	0.011	0.004-0.099	0.3013	0.3
**Alistipes_finegoldii**	0.007	0.001-0.028	0.031	0.001-0.069	0.2664	0.27
**X.Eubacterium._cellulosolvens**	0.007	0.001-0.012	0.007	0.003-0.013	0.6221	0.62
**Bacteroides_helcogenes**	0.007	0-0.011	0.012	0.005-0.051	0.1514	0.15
**Bifidobacterium_catenulatum**	0.006	0.001-0.104	0.003	0.001-0.036	0.4697	0.47
**Ruminococcus_bicirculans**	0.006	0.001-0.06	0.003	0.001-0.021	0.6772	0.68
**Eubacterium_maltosivorans**	0.006	0.002-0.014	0.009	0.005-0.011	0.9697	0.97
**Clostridium_perfringens**	0.006	0.002-0.01	0.005	0.002-0.02	0.2661	0.27

**Clostridium_botulinum**	0.006	0.002-0.008	0.006	0.002-0.015	0.3394	0.34
**Haemophilus_parainfluenzae**	0.006	0-0.059	0.013	0.001-0.059	0.8501	0.85
**Monoglobus_pectinilyticus**	0.005	0.003-0.009	0.008	0.002-0.013	0.6221	0.62
**Clostridium_sporogenes**	0.005	0.001-0.014	0.008	0.004-0.019	0.5186	0.52
**Akkermansia_muciniphila**	0.004	0.002-0.01	0.007	0.003-0.088	0.5693	0.57
**Streptococcus_thermophilus**	0.004	0.002-0.008	0.003	0.001-0.013	0.9097	0.91
**Collinsella_aerofaciens**	0.004	0-0.039	0.003	0.001-0.085	0.9697	0.97
**Enterobacter_cloacae**	0.004	0.001-0.007	0.002	0.001-0.005	0.5186	0.52
**Streptococcus_pasteurianus**	0.004	0.002-0.016	0.01	0.004-0.052	0.2334	0.23
**Eubacterium_callanderi**	0.004	0.001-0.026	0.005	0.003-0.016	0.5186	0.52
**Bifidobacterium_adolescentis**	0.004	0.001-0.028	0.001	0.001-0.01	0.5186	0.52
**Bacteroides_heparinolyticus**	0.004	0-0.005	0.006	0.003-0.026	0.2036	0.2
**Prevotella_intermedia**	0.003	0-0.006	0.006	0.003-0.008	0.3804	0.38
**Ruminococcus_champanellensis**	0.003	0-0.011	0.003	0.001-0.008	0.7334	0.73
**Bacteroides_zoogleoformans**	0.003	0-0.006	0.006	0.003-0.023	0.2661	0.27
**Clostridium_butyricum**	0.003	0.001-0.007	0.004	0.002-0.01	0.3394	0.34
**Gordonibacter_pamelaeae**	0.002	0.001-0.008	0.002	0.001-0.006	0.7334	0.73
**Ruminococcus_sp._JE7A12**	0.002	0.001-0.008	0.002	0.001-0.006	0.9697	0.97
**Clostridium_saccharoperbutylacetonicum**	0.002	0.001-0.004	0.002	0.001-0.007	0.4697	0.47
**Clostridium_baratii**	0.002	0.001-0.004	0.002	0.001-0.006	0.4697	0.47
**Megasphaera_elsdenii**	0.002	0-0.004	0.002	0.001-0.003	0.8501	0.85
**Alistipes_sp._6CPBBH3**	0.002	0-0.009	0.007	0-0.026	0.4498	0.45
**Bacillus_cereus**	0.002	0.001-0.119	0.004	0.001-0.056	0.2661	0.27
**Prevotella_dentalis**	0.002	0-0.003	0.002	0.001-0.004	0.5186	0.52
**Barnesiella_viscericola**	0.002	0.001-0.006	0.012	0.003-0.022	0.0024	0.0024
**Acidaminococcus_fermentans**	0.002	0-0.006	0.002	0.001-0.005	0.6221	0.62
**Alistipes_shahii**	0.002	0.001-0.017	0.006	0.001-0.04	0.0342	0.034
**Alistipes_sp._Marseille_P5997**	0.002	0-0.003	0.004	0.001-0.012	0.083	0.083
**Enterococcus_avium**	0.002	0.001-0.007	0.002	0.001-0.005	1	1
**Alistipes_sp._3BBH6**	0.001	0-0.002	0	0-0.006	0.3066	0.31
**Bifidobacterium_dentium**	0.001	0-0.003	0	0-0.003	0.3013	0.3
**Fusobacterium_mortiferum**	0.001	0-0.001	0.001	0-0.002	0.1099	0.11
**Acidaminococcus_intestini**	0.001	0-0.001	0.001	0-0.003	0.4697	0.47
**Alistipes_sp._5CPEGH6**	0.001	0-0.014	0.016	0-0.031	0.0122	0.012
**Bacteroides_coprosuis**	0.001	0-0.001	0.003	0.001-0.007	0.0049	0.0049
**Megamonas_hypermegale**	0.001	0.001-0.003	0.001	0.001-0.002	0.6221	0.62
**Alistipes_sp._5CBH24**	0.001	0-0.003	0.003	0-0.009	0.0367	0.037
**Morganella_morganii**	0.001	0.001-0.053	0.002	0.001-0.054	0.4238	0.42
**Klebsiella_variicola**	0.001	0.001-0.007	0.001	0-0.006	0.6772	0.68
**Prevotella_fusca**	0.001	0-0.001	0.001	0-0.001	0.7334	0.73
**Prevotella_ruminicola**	0.001	0-0.002	0.002	0.001-0.004	0.0923	0.092
**Prevotella_melaninogenica**	0.001	0.001-0.002	0.001	0.001-0.003	0.6221	0.62

**Actinoalloteichus_sp._AHMU_CJ021**	0	0-0.191	0	0-0.09	0.1099	0.11
**Bifidobacterium_animalis**	0	0-0.002	0.001	0-0.002	0.8501	0.85
**Prevotella_oris**	0	0-0.001	0.001	0-0.002	0.791	0.79
**Prevotella_denticola**	0	0-0.002	0.001	0-0.003	0.5693	0.57
**Phascolarctobacterium_succinatutens**	0	0-0.001	0.001	0-0.014	0.8939	0.89
**uncultured_crAssphage**	0	0-0.004	0	0-0.001	0.2945	0.29
**Chryseobacterium_taklimakanense**	0	0-0.001	0.001	0-0.002	0.1099	0.11
**Phascolarctobacterium_faecium**	0	0-0	0	0-0.015	0.7557	0.76

Pre_KD, samples collected before ketogenic diet initiation; Post_KD, samples collected after one week of ketogenic diet therapy.

TABLE S8 Paired comparison between Pre_KD and Post_KD groups at KEGG pathway

KEGG Pathway	Pre_KD (median)	Pre_KD (Q1-Q3)	Post_KD (median)	Post_KD (Q1-Q3)	Pre_KD_vs_Post_KD (p.value)	Pre_KD_vs_Post_KD (p.adj)
Metabolic_pathways	16.548	16.006-17.065	17.037	16-17.119	0.5693	0.57
Biosynthesis_of_secondary_metabolites	6.855	6.748-6.888	6.702	6.391-6.814	0.1514	0.15
Biosynthesis_of_antibiotics	5.118	5.055-5.225	4.998	4.865-5.151	0.0771	0.077
Microbial_metabolism_in_diverse_environments	4.579	4.167-4.774	4.276	4.093-4.488	0.0923	0.092
Biosynthesis_of_amino_acids	3.456	3.328-3.626	3.327	3.236-3.474	0.1514	0.15
ABC_transporters	3.074	2.203-3.505	2.058	1.702-2.423	0.0122	0.012
Two_component_system	2.599	2.183-2.76	2.421	2.259-2.79	0.7334	0.73
Carbon_metabolism	2.476	2.448-2.539	2.509	2.445-2.576	0.7334	0.73
Purine_metabolism	1.981	1.928-2.039	1.977	1.919-2.043	0.4697	0.47
Pyrimidine_metabolism	1.551	1.491-1.702	1.52	1.455-1.612	0.6221	0.62
Ribosome	1.537	1.409-1.712	1.473	1.407-1.537	0.2036	0.2
Amino_sugar_and_nucleotide_sugar_metabolism	1.454	1.243-1.741	1.708	1.417-1.879	0.0024	0.0024
Starch_and_sucrose_metabolism	1.313	1.095-1.461	1.311	1.105-1.516	0.4697	0.47
Galactose_metabolism	1.153	0.95-1.333	1.167	0.878-1.259	0.7334	0.73
Glycolysis__Gluconeogenesis	1.112	1.091-1.182	1.16	1.104-1.187	0.5693	0.57
Pyruvate_metabolism	1.005	0.947-1.033	0.999	0.971-1.055	0.9697	0.97
Cysteine_and_methionine_metabolism	0.929	0.906-0.957	0.9	0.826-0.947	0.2036	0.2
Alanine_aspartate_and_glutamate_metabolism	0.851	0.804-0.889	0.864	0.746-0.917	0.5693	0.57
Fructose_and_mannose_metabolism	0.835	0.781-0.915	0.86	0.846-0.934	0.0923	0.092
Oxidative_phosphorylation	0.829	0.744-0.86	0.834	0.789-0.893	0.5693	0.57
Glycine_serine_and_threonine_metabolism	0.815	0.745-0.858	0.764	0.738-0.781	0.0161	0.016
Homologous_recombination	0.811	0.782-0.826	0.77	0.718-0.846	0.2661	0.27
Carbon_fixation_pathways_in_prokaryotes	0.808	0.753-0.845	0.813	0.79-0.856	0.5693	0.57
Aminoacyl_tRNA_biosynthesis	0.807	0.722-0.857	0.695	0.662-0.833	0.1514	0.15
Phosphotransferase_system_PTS	0.788	0.484-0.921	0.433	0.295-1.13	0.9697	0.97
Pentose_phosphate_pathway	0.753	0.721-0.794	0.754	0.725-0.78	0.8501	0.85
xocarboxylic_acid_metabolism	0.752	0.702-0.783	0.681	0.628-0.704	0.0161	0.016
Methane_metabolism	0.724	0.713-0.733	0.722	0.649-0.736	0.5693	0.57
Glyoxylate_and_dicarboxylate_metabolism	0.719	0.68-0.764	0.703	0.679-0.73	0.2334	0.23
Bacterial_secretion_system	0.69	0.539-0.819	0.55	0.5-0.658	0.0522	0.052
Mismatch_repair	0.673	0.635-0.714	0.691	0.66-0.734	0.9697	0.97
Peptidoglycan_biosynthesis	0.635	0.589-0.674	0.56	0.526-0.662	0.1763	0.18
Propanoate_metabolism	0.619	0.571-0.667	0.591	0.533-0.659	0.4697	0.47
DNA_replication	0.612	0.588-0.639	0.646	0.594-0.721	0.1514	0.15
Butanoate_metabolism	0.592	0.511-0.686	0.526	0.493-0.594	0.2334	0.23
Porphyrin_and_chlorophyll_metabolism	0.591	0.542-0.662	0.612	0.545-0.677	0.791	0.79

**Pentose_and_glucuronate_interconversions**	0.588	0.518-0.615	0.529	0.477-0.592	0.9097	0.91
**Phenylalanine_tyrosine_and_tryptophan_biosynthesis**	0.568	0.524-0.609	0.562	0.531-0.596	0.4697	0.47
**RNA_degradation**	0.562	0.521-0.585	0.582	0.484-0.625	0.7334	0.73
**Citrate_cycle_TCA_cycle**	0.526	0.482-0.573	0.567	0.497-0.611	0.7334	0.73
**Glycerophospholipid_metabolism**	0.519	0.496-0.532	0.473	0.439-0.519	0.064	0.064
**beta_Lactam_resistance**	0.505	0.443-0.605	0.642	0.456-0.693	0.5693	0.57
**Fatty_acid_metabolism**	0.486	0.451-0.51	0.501	0.482-0.546	0.0771	0.077
**Lysine_biosynthesis**	0.48	0.467-0.499	0.465	0.441-0.48	0.3013	0.3
**Arginine_biosynthesis**	0.476	0.447-0.494	0.468	0.411-0.496	0.4238	0.42
**Pantothenate_and_CoA_biosynthesis**	0.465	0.423-0.493	0.445	0.402-0.461	0.1763	0.18
**One_carbon_pool_by_folate**	0.461	0.43-0.489	0.473	0.45-0.484	0.2661	0.27
**Carbon_fixation_in_photosynthetic_organisms**	0.454	0.435-0.468	0.473	0.456-0.487	0.0522	0.052
**Protein_export**	0.44	0.413-0.468	0.444	0.422-0.451	0.8501	0.85
**Nicotinate_and_nicotinamide_metabolism**	0.427	0.402-0.446	0.402	0.352-0.427	0.0425	0.042
**Valine_leucine_and_isoleucine_biosynthesis**	0.422	0.352-0.469	0.346	0.321-0.385	0.0093	0.0093
**other**	0.418	0.321-0.457	0.418	0.317-0.436	0.9697	0.97
**Cationic_antimicrobial_peptide_CAMP_resistance**	0.41	0.338-0.466	0.444	0.353-0.499	0.3394	0.34
**Fatty_acid_biosynthesis**	0.405	0.376-0.459	0.467	0.424-0.506	0.021	0.021
**Folate_biosynthesis**	0.402	0.355-0.431	0.368	0.315-0.421	0.7334	0.73
**Other_glycan_degradation**	0.399	0.285-0.881	0.678	0.352-0.826	0.1099	0.11
**Nitrogen_metabolism**	0.391	0.368-0.473	0.373	0.36-0.437	0.2661	0.27
**Streptomycin_biosynthesis**	0.39	0.352-0.452	0.406	0.359-0.47	0.2334	0.23
**Cell_cycle___Caulobacter**	0.39	0.371-0.419	0.411	0.379-0.453	0.5186	0.52
**Arginine_and_proline_metabolism**	0.388	0.36-0.425	0.354	0.34-0.379	0.0425	0.042
**Selenocompound_metabolism**	0.377	0.353-0.4	0.341	0.308-0.379	0.0522	0.052
**Sulfur_metabolism**	0.368	0.288-0.423	0.337	0.322-0.37	0.5693	0.57
**Thiamine_metabolism**	0.352	0.318-0.392	0.372	0.309-0.395	1	1
**Histidine_metabolism**	0.346	0.318-0.377	0.345	0.311-0.38	0.8501	0.85
**Nucleotide_excision_repair**	0.343	0.312-0.359	0.356	0.331-0.38	0.3013	0.3
**Glycerolipid_metabolism**	0.342	0.316-0.351	0.353	0.317-0.406	0.3394	0.34
**Base_excision_repair**	0.34	0.334-0.359	0.363	0.328-0.379	0.2661	0.27
**Terpenoid_backbone_biosynthesis**	0.325	0.317-0.346	0.318	0.298-0.335	0.5693	0.57
**Lipopolysaccharide_biosynthesis**	0.323	0.201-0.4	0.32	0.248-0.384	0.9097	0.91
**Biotin_metabolism**	0.315	0.276-0.358	0.346	0.3-0.369	0.7334	0.73
**Endocytosis**	0.31	0.144-0.437	0.336	0.121-0.584	0.6221	0.62
**Bacterial_chemotaxis**	0.307	0.255-0.331	0.349	0.251-0.422	0.4238	0.42
**Sphingolipid_metabolism**	0.284	0.183-0.597	0.431	0.245-0.642	0.2036	0.2
**Sulfur_relay_system**	0.277	0.252-0.307	0.234	0.215-0.264	0.0522	0.052
**Photosynthesis**	0.257	0.222-0.292	0.222	0.204-0.249	0.1763	0.18

**Degradation_of_aromatic_compounds**	0.254	0.155-0.286	0.155	0.144-0.206	0.1294	0.13
**Phenylalanine_metabolism**	0.252	0.213-0.31	0.226	0.197-0.245	0.064	0.064
**Drug_metabolism___other_enzymes**	0.247	0.23-0.272	0.266	0.252-0.283	0.0342	0.034
**Valine_leucine_and_isoleucine_degradation**	0.246	0.215-0.275	0.262	0.235-0.281	0.9697	0.97
**Flagellar_assembly**	0.245	0.122-0.294	0.129	0.116-0.226	0.5693	0.57
**Vancomycin_resistance**	0.244	0.221-0.275	0.278	0.216-0.34	0.7334	0.73
**Fatty_acid_degradation**	0.241	0.204-0.281	0.195	0.181-0.24	0.3013	0.3
**Polyketide_sugar_unit_biosynthesis**	0.236	0.198-0.271	0.196	0.18-0.254	0.8501	0.85
**C5_Branched_dibasic_acid_metabolism**	0.232	0.216-0.28	0.216	0.198-0.241	0.0342	0.034
**Cyanoamino_acid_metabolism**	0.228	0.189-0.305	0.294	0.196-0.349	0.0923	0.092
**Ubiquinone_and_other_terpenoid_quinone_biosynthesis**	0.225	0.158-0.27	0.201	0.162-0.235	0.3804	0.38
**Glutathione_metabolism**	0.222	0.182-0.246	0.186	0.152-0.196	0.021	0.021
**Amoebiasis**	0.221	0.159-0.311	0.205	0.144-0.407	0.9097	0.91
**Monobactam_biosynthesis**	0.218	0.205-0.223	0.206	0.186-0.216	0.1514	0.15
**Protein_digestion_and_absorption**	0.216	0.188-0.259	0.212	0.153-0.243	1	1
**Tyrosine_metabolism**	0.202	0.18-0.269	0.166	0.148-0.211	0.0269	0.027
**Vitamin_B6_metabolism**	0.188	0.172-0.198	0.175	0.138-0.194	0.2036	0.2
**Phenylpropanoid_biosynthesis**	0.184	0.122-0.231	0.228	0.16-0.292	0.0269	0.027
**Peroxisome**	0.175	0.165-0.179	0.179	0.143-0.199	0.9697	0.97
**Benzoate_degradation**	0.172	0.12-0.251	0.12	0.108-0.177	0.0522	0.052
**Central_carbon_metabolism_in_cancer**	0.169	0.14-0.189	0.166	0.142-0.187	0.8501	0.85
**Vibrio_cholerae_infection**	0.164	0.111-0.279	0.128	0.071-0.191	0.4238	0.42
**Riboflavin_metabolism**	0.164	0.138-0.189	0.17	0.12-0.184	0.9097	0.91
**Longevity_regulating_pathway___worm**	0.158	0.147-0.177	0.16	0.148-0.175	0.9697	0.97
**Ascorbate_and_aldarate_metabolism**	0.157	0.121-0.217	0.136	0.122-0.16	0.3013	0.3
**Inositol_phosphate_metabolism**	0.151	0.108-0.175	0.18	0.163-0.208	0.0068	0.0068
**RNA_polymerase**	0.151	0.138-0.17	0.15	0.124-0.159	0.2661	0.27
**Pertussis**	0.14	0.072-0.157	0.118	0.075-0.147	0.6772	0.68
**Glucagon_signaling_pathway**	0.136	0.118-0.168	0.131	0.107-0.147	0.1763	0.18
**RNA_transport**	0.136	0.114-0.157	0.156	0.139-0.187	0.1763	0.18
**Lysine_degradation**	0.133	0.099-0.189	0.11	0.095-0.199	0.791	0.79
**Acarbose_and_validamycin_biosynthesis**	0.13	0.113-0.166	0.125	0.101-0.141	0.3804	0.38
**Biosynthesis_of_unsaturated_fatty_acids**	0.127	0.114-0.132	0.129	0.118-0.137	1	1
**Lysosome**	0.125	0.04-0.368	0.324	0.152-0.51	0.0161	0.016
**beta_Alanine_metabolism**	0.123	0.097-0.174	0.124	0.106-0.144	0.5693	0.57
**Tuberculosis**	0.12	0.112-0.151	0.116	0.103-0.128	0.6221	0.62
**Longevity_regulating_pathway___multiple_species**	0.119	0.095-0.237	0.215	0.084-0.287	0.3804	0.38
**Aminobenzoate_degradation**	0.118	0.096-0.128	0.099	0.087-0.108	0.0923	0.092

**Chloroalkane_and_chloroalkene_degradation**	0.118	0.08-0.131	0.074	0.044-0.116	0.1514	0.15
**Legionellosis**	0.115	0.098-0.124	0.106	0.101-0.119	0.2036	0.2
**Novobiocin_biosynthesis**	0.112	0.1-0.115	0.103	0.101-0.114	0.3804	0.38
**PPAR_signaling_pathway**	0.112	0.083-0.126	0.119	0.07-0.13	0.5693	0.57
**Phosphonate_and_phosphinate_metabolism**	0.111	0.104-0.135	0.097	0.09-0.104	0.791	0.79
**D_Glutamine_and_D_glutamate_metabolism**	0.11	0.103-0.119	0.11	0.093-0.12	0.5693	0.57
**Taurine_and_hypotaurine_metabolism**	0.106	0.1-0.121	0.104	0.094-0.114	0.2334	0.23
**Salmonella_infection**	0.106	0.09-0.135	0.103	0.084-0.222	0.6221	0.62
**Tropane_piperidine_and_pyridine_alkaloid_biosynthesis**	0.104	0.09-0.112	0.097	0.083-0.104	0.0522	0.052
**Glycosaminoglycan_degradation**	0.101	0.067-0.295	0.28	0.135-0.402	0.0342	0.034
**Glycosphingolipid_biosynthesis___globo_series**	0.099	0.068-0.258	0.23	0.117-0.293	0.0269	0.027
**Tryptophan_metabolism**	0.097	0.064-0.121	0.078	0.068-0.097	0.2661	0.27
**GABAergic_synapse**	0.095	0.089-0.112	0.105	0.086-0.115	0.1099	0.11
**Insulin_resistance**	0.094	0.079-0.114	0.09	0.077-0.098	0.3013	0.3
**Plant_pathogen_interaction**	0.091	0.079-0.103	0.109	0.098-0.12	0.0923	0.092
**Butirosin_and_neomycin_biosynthesis**	0.088	0.063-0.098	0.09	0.068-0.113	0.3394	0.34
**Tetracycline_biosynthesis**	0.085	0.067-0.096	0.069	0.06-0.091	0.2334	0.23
**MicroRNAs_in_cancer**	0.085	0.07-0.102	0.092	0.084-0.122	0.4697	0.47
**Adipocytokine_signaling_pathway**	0.081	0.054-0.093	0.09	0.034-0.099	0.9697	0.97
**Naphthalene_degradation**	0.081	0.058-0.097	0.051	0.034-0.085	0.1294	0.13
**D_Alanine_metabolism**	0.081	0.078-0.083	0.07	0.064-0.082	0.1294	0.13
**Biosynthesis_of_ansamycins**	0.078	0.066-0.083	0.075	0.066-0.086	0.6221	0.62
**Protein_processing_in_endoplasmic_reticulum**	0.078	0.043-0.093	0.072	0.066-0.083	0.9097	0.91
**Glutamatergic_synapse**	0.076	0.067-0.085	0.084	0.068-0.097	0.1099	0.11
**Metabolism_of_xenobiotics_by_cytochrome_P450**	0.076	0.051-0.093	0.053	0.043-0.066	0.0522	0.052
**HIF_1_signaling_pathway**	0.075	0.071-0.088	0.079	0.071-0.084	0.6772	0.68
**mRNA_surveillance_pathway**	0.074	0.055-0.101	0.073	0.056-0.121	0.3804	0.38
**Insulin_signaling_pathway**	0.072	0.067-0.078	0.07	0.064-0.074	0.9697	0.97
**AMPK_signaling_pathway**	0.071	0.042-0.081	0.069	0.065-0.09	0.3013	0.3
**Phosphatidylinositol_signaling_system**	0.07	0.065-0.079	0.07	0.061-0.08	0.8501	0.85
**Bacterial_invasion_of_epithelial_cells**	0.069	0.057-0.08	0.098	0.053-0.165	0.2661	0.27
**Huntingtons_disease**	0.069	0.059-0.106	0.058	0.054-0.082	0.2334	0.23
**Vibrio_cholerae_pathogenic_cycle**	0.068	0.054-0.087	0.07	0.051-0.082	0.791	0.79
**Epithelial_cell_signaling_in_Helicobacter_pylori_infection**	0.066	0.06-0.073	0.062	0.055-0.07	0.1099	0.11
**Nitrotoluene_degradation**	0.063	0.052-0.088	0.062	0.052-0.087	1	1
**Carbapenem_biosynthesis**	0.061	0.053-0.065	0.054	0.051-0.059	0.1514	0.15
**Biosynthesis_of_siderophore_group_nonribosomal_peptides**	0.057	0.037-0.083	0.037	0.025-0.042	0.1294	0.13
**Drug_metabolism___cytochrome_P450**	0.057	0.036-0.076	0.029	0.024-0.037	0.0522	0.052

**Retinol_metabolism**	0.055	0.045-0.059	0.033	0.027-0.042	0.0522	0.052
**Isoquinoline_alkaloid_biosynthesis**	0.054	0.048-0.06	0.049	0.045-0.051	0.1099	0.11
**Choline_metabolism_in_cancer**	0.054	0.045-0.072	0.065	0.038-0.136	0.4238	0.42
**Type_I_diabetes_mellitus**	0.051	0.046-0.056	0.049	0.041-0.052	0.4238	0.42
**Meiosis___yeast**	0.05	0.009-0.172	0.142	0.014-0.204	0.2661	0.27
**Platinum_drug_resistance**	0.047	0.024-0.062	0.021	0.015-0.032	0.0342	0.034
**Pathways_in_cancer**	0.047	0.038-0.061	0.05	0.047-0.055	0.791	0.79
**PI3K_Akt_signaling_pathway**	0.047	0.036-0.066	0.064	0.053-0.081	0.3804	0.38
**Biosynthesis_of_vancomycin_group_antibiotics**	0.045	0.04-0.053	0.038	0.03-0.048	0.1763	0.18
**Fc_gamma_R_mediated_phagocytosis**	0.044	0.03-0.049	0.029	0.021-0.127	0.6772	0.68
**N_Glycan_biosynthesis**	0.044	0.026-0.056	0.06	0.041-0.07	0.064	0.064
**Alzheimers_disease**	0.042	0.039-0.052	0.042	0.035-0.046	0.1294	0.13
**Pathogenic_Escherichia_coli_infection**	0.042	0.022-0.062	0.06	0.03-0.122	0.3394	0.34
**Ribosome_biogenesis_in_eukaryotes**	0.042	0.04-0.05	0.035	0.03-0.043	0.064	0.064
**Regulation_of_actin_cytoskeleton**	0.041	0.02-0.062	0.03	0.024-0.125	0.5693	0.57
**Shigellosis**	0.039	0.018-0.048	0.029	0.019-0.126	0.6221	0.62
**Limonene_and_pinene_degradation**	0.038	0.024-0.054	0.021	0.012-0.036	0.0522	0.052
**Secondary_bile_acid_biosynthesis**	0.037	0.021-0.052	0.049	0.035-0.058	0.0425	0.042
**Type_II_diabetes_mellitus**	0.037	0.033-0.041	0.039	0.033-0.047	0.4238	0.42
**Lipoic_acid_metabolism**	0.036	0.032-0.042	0.039	0.026-0.057	0.3013	0.3
**Spliceosome**	0.036	0.019-0.052	0.028	0.018-0.053	0.9097	0.91
**AGE_RAGE_signaling_pathway_in_diabetic_complications**	0.036	0.022-0.055	0.048	0.033-0.089	0.4697	0.47
**Progesterone_mediated_oocyte_maturation**	0.036	0.028-0.04	0.039	0.034-0.045	0.2661	0.27
**Adherens_junction**	0.036	0.018-0.056	0.028	0.02-0.122	0.6221	0.62
**Chemokine_signaling_pathway**	0.035	0.017-0.046	0.026	0.019-0.121	0.6221	0.62
**Penicillin_and_cephalosporin_biosynthesis**	0.035	0.028-0.056	0.044	0.032-0.051	0.4238	0.42
**Primary_immunodeficiency**	0.035	0.027-0.036	0.038	0.028-0.045	0.1099	0.11
**Glycosphingolipid_biosynthesis___ganglio_series**	0.035	0.008-0.152	0.14	0.064-0.199	0.0122	0.012
**Dorso_ventral_axis_formation**	0.035	0.019-0.052	0.028	0.021-0.083	0.9097	0.91
**Proteoglycans_in_cancer**	0.033	0.03-0.038	0.029	0.027-0.035	0.0771	0.077
**Viral_carcinogenesis**	0.033	0.032-0.039	0.033	0.03-0.042	0.5693	0.57
**FoxO_signaling_pathway**	0.032	0.028-0.038	0.036	0.029-0.042	0.6221	0.62
**Apoptosis___fly**	0.031	0.028-0.043	0.029	0.025-0.043	0.5693	0.57
**ECM_receptor_interaction**	0.03	0.021-0.041	0.035	0.013-0.051	0.6221	0.62
**Primary_bile_acid_biosynthesis**	0.03	0.018-0.05	0.04	0.024-0.057	0.0923	0.092
**Zeatin_biosynthesis**	0.03	0.028-0.041	0.04	0.027-0.047	0.3013	0.3
**Synthesis_and_degradation_of_ketone_bodies**	0.03	0.012-0.04	0.017	0.009-0.042	0.5186	0.52
**Epstein_Barr_virus_infection**	0.029	0.02-0.046	0.026	0.021-0.034	0.5186	0.52
**Herpes_simplex_infection**	0.029	0.02-0.047	0.027	0.02-0.035	0.5693	0.57

**Amyotrophic_lateral_sclerosis_ALS**	0.028	0.017-0.039	0.028	0.019-0.034	0.6772	0.68
**Focal_adhesion**	0.028	0.02-0.043	0.034	0.013-0.079	0.5693	0.57
**NOD_like_receptor_signaling_pathway**	0.028	0.018-0.041	0.032	0.028-0.037	0.2661	0.27
**Longevity_regulating_pathway**	0.028	0.024-0.036	0.032	0.026-0.041	0.791	0.79
**Platelet_activation**	0.027	0.015-0.033	0.035	0.017-0.067	0.5186	0.52
**Antigen_processing_and_presentation**	0.026	0.017-0.032	0.028	0.026-0.032	0.3013	0.3
**Geraniol_degradation**	0.024	0.007-0.038	0.011	0.004-0.027	0.3013	0.3
**Proximal_tubule_bicarbonate_reclamation**	0.024	0.014-0.031	0.025	0.02-0.033	0.4697	0.47
**MAPK_signaling_pathway___yeast**	0.024	0.021-0.032	0.026	0.023-0.03	0.7334	0.73
**Estrogen_signaling_pathway**	0.023	0.016-0.03	0.027	0.025-0.031	0.0923	0.092
**Prostate_cancer**	0.023	0.017-0.03	0.028	0.025-0.031	0.0923	0.092
**Chlorocyclohexane_and_chlorobenzene_degradation**	0.023	0.013-0.026	0.012	0.008-0.021	0.2036	0.2
**Carbohydrate_digestion_and_absorption**	0.023	0.018-0.03	0.027	0.021-0.039	0.4697	0.47
**Xylene_degradation**	0.023	0.014-0.041	0.012	0.009-0.022	0.021	0.021
**Thyroid_hormone_synthesis**	0.022	0.016-0.027	0.018	0.012-0.028	0.791	0.79
**Steroid_hormone_biosynthesis**	0.022	0.011-0.06	0.04	0.012-0.074	0.4697	0.47
**MAPK_signaling_pathway___fly**	0.021	0.016-0.022	0.022	0.016-0.025	1	1
**Dioxin_degradation**	0.021	0.01-0.034	0.01	0.006-0.017	0.0425	0.042
**Prolactin_signaling_pathway**	0.02	0.014-0.03	0.014	0.006-0.016	0.021	0.021
**Chemical_carcinogenesis**	0.019	0.008-0.04	0.01	0.004-0.014	0.1514	0.15
**Caprolactam_degradation**	0.018	0.008-0.029	0.023	0.011-0.029	0.5186	0.52
**Plant_hormone_signal_transduction**	0.018	0.002-0.029	0.014	0.006-0.025	0.9697	0.97
**Nonribosomal_peptide_structures**	0.017	0.011-0.021	0.016	0.011-0.02	0.5186	0.52
**Styrene_degradation**	0.017	0.012-0.029	0.013	0.009-0.022	0.4697	0.47
**Ether_lipid_metabolism**	0.017	0.011-0.021	0.015	0.008-0.025	0.791	0.79
**Arachidonic_acid_metabolism**	0.017	0.011-0.025	0.01	0.007-0.02	0.1763	0.18
**Biosynthesis_of_type_II_polyketide_products**	0.014	0.008-0.018	0.008	0.006-0.012	0.3013	0.3
**Salivary_secretion**	0.014	0.006-0.063	0.026	0.01-0.042	0.9097	0.91
**Flavone_and_flavonol_biosynthesis**	0.014	0.009-0.02	0.018	0.008-0.023	0.3394	0.34
**Stilbenoid_diarylheptanoid_and_gingerol_biosynthesis**	0.013	0.005-0.021	0.019	0.012-0.024	0.064	0.064
**Flavonoid_biosynthesis**	0.013	0.005-0.021	0.019	0.012-0.024	0.064	0.064
**Phospholipase_D_signaling_pathway**	0.013	0.009-0.016	0.015	0.008-0.018	0.3804	0.38
**Cell_cycle___yeast**	0.013	0.011-0.015	0.012	0.006-0.018	0.791	0.79
**Cell_cycle**	0.012	0.007-0.015	0.012	0.005-0.018	0.8501	0.85
**Ubiquitin_mediated_proteolysis**	0.012	0.007-0.015	0.012	0.005-0.018	0.8501	0.85
**Calcium_signaling_pathway**	0.01	0.007-0.013	0.014	0.006-0.02	0.7334	0.73
**Small_cell_lung_cancer**	0.008	0.004-0.022	0.007	0.006-0.01	0.9697	0.97
**Staphylococcus_aureus_infection**	0.007	0.007-0.014	0.01	0.004-0.02	0.4697	0.47

**Apoptosis**	0.006	0.002-0.026	0.026	0.013-0.036	0.0049	0.0049
**GlycosylphosphatidylinositolGPI_anchor_biosynthesis**	0.004	0.001-0.019	0.005	0.001-0.01	0.8501	0.85
**Rheumatoid_arthritis**	0.003	0.001-0.013	0.005	0.002-0.015	0.6891	0.69
**Tight_junction**	0.002	0-0.007	0.002	0.001-0.004	0.6891	0.69
**Arrhythmogenic_right_ventricular_cardiomyopathy_ARVC**	0.002	0.001-0.009	0.004	0.001-0.007	0.5693	0.57
**Osteoclast_differentiation**	0.001	0-0.01	0.005	0.002-0.014	0.2664	0.27
**Glycosphingolipid_biosynthesis___lacto_and_neolacto_series**	0.001	0-0.006	0.002	0.001-0.006	0.7557	0.76
**Systemic_lupus_erythematosus**	0	0-0	0	0-0	1	1
**Leukocyte_transendothelial_migration**	0	0-0.004	0	0-0.003	1	1

Pre_KD, samples collected before ketogenic diet initiation; Post_KD, samples collected after one week of ketogenic diet therapy.

TABLE S9 Paired comparison between Pre_KD and Post_KD groups at metabolites

Metabolites	Pre_KD (median)	Pre_KD (Q1-Q3)	Post_KD (median)	Post_KD (Q1-Q3)	Pre_KD_vs_Post_KD (p.value)	Pre_KD_vs_Post_KD (p.adj)	log2foldchange
Deoxyguanosine	386.282	284.878-514.938	222.555	180.577-274.229	0.0391	0.039	0.795492539
Epidermin	374.911	191.039-661.135	33.235	4.582-54.037	0.0156	0.016	3.495772896
Hydroxyprolyl-Leucine	336.221	75.658-847.791	3.291	2.145-9.716	0.0078	0.0078	6.67474
Lutein	132.332	66.703-394.783	37.263	14.176-67.781	0.0078	0.0078	1.828346237
Palmitoyl Serinol	107.548	27.794-278.782	27.857	15.461-65.576	0.0078	0.0078	1.948868893
4-Guanidinobutanoic acid	95.686	23.394-204.624	29.894	17.265-45.463	0.0234	0.023	1.678451905
Heptane-1-thiol	63.131	31.296-169.325	10.899	6.721-17.387	0.0234	0.023	2.534152832
D-1-Piperideine-2-carboxylic acid	58.669	36.365-120.147	23.124	12.118-25.724	0.0156	0.016	1.343207424
PC(18:0/18:2(9Z,12Z))	48.761	16.222-172.241	12.902	1.555-73.637	0.0078	0.0078	1.918132991
beta-Cryptoxanthin	46.614	23.43-86.001	11.382	3.453-16.927	0.0078	0.0078	2.034009233
Triethanolamine	46.612	13.894-336.467	5.244	4.025-19.721	0.0078	0.0078	3.151961826
Norvaline	37.733	7.528-80.745	5.813	3.7-7.49	0.0234	0.023	2.698471994
Ginsenoyne B	33.943	2.581-130.279	1.066	0.001-8.292	0.0156	0.016	4.992834738
Prolyl-Valine	33.591	12.492-132.111	5.231	4.355-13.501	0.0156	0.016	2.68291607
4-Aminobutyraldehyde	25.824	10.03-46.562	12.543	10.049-14.595	0.0391	0.039	1.041830035
Isoquinoline	25.129	19.324-72.743	0.342	0.001-2.413	0.0391	0.039	6.199213125
(+)-2,3-Dihydro-3-methyl-1H-pyrrole	19.837	4.778-62.253	3.234	2.583-4.124	0.0078	0.0078	2.616802276
Trimethylamine N-oxide	18.646	6.491-34.176	7.117	2.496-20.45	0.0391	0.039	1.389525031
Uracil	17.984	13.772-32.107	4.537	3.154-7.177	0.0078	0.0078	1.986903376
2(N)-Methyl-norsalsolinol	17.453	9.877-109.714	6.622	2.588-9.18	0.0234	0.023	1.398136127
Jasmolone	15.2	8.307-40.951	1.89	1.366-3.344	0.0078	0.0078	3.007613184
Indole-3-carboxylic acid	15.102	12.18-22.417	2.433	1.744-14.226	0.0234	0.023	2.633931397
Nicotinic acid mononucleotide	14.477	7.47-20.611	3.122	2.222-5.182	0.0078	0.0078	2.213220228
Laccarin	14.291	6.218-50.916	5.2	2.4-13.662	0.0078	0.0078	1.458523343
Alanyl-Proline	14.058	3.195-31.808	3.25	1.856-6.06	0.0391	0.039	2.112879737
N-Carboxyethyl-g-aminobutyric acid	13.271	5.579-40.214	2.758	2.272-4.618	0.0078	0.0078	2.266582723
L-Isoleucine	11.549	2.966-21.733	3.975	3.072-7.114	0.0391	0.039	1.538741172
LysoPC(18:2(9Z,12Z))	11.088	0.513-21.751	0.37	0.157-0.717	0.0391	0.039	4.905330082
Pyrophaeophorbide a	10.05	4.774-63.185	0.948	0.674-1.314	0.0078	0.0078	3.406164632
3-Formyl-6-hydroxyindole	9.078	4.337-81.615	1.652	0.66-6.313	0.0078	0.0078	2.458160802
Ursodeoxycholic acid	8.63	6.137-18.863	1.12	0.606-3.462	0.0078	0.0078	2.945861827
trans-S-(1-Propenyl)-L-cysteine	8.37	1.851-53.7	1.355	0.921-2.848	0.0234	0.023	2.626934771
Isovalerylglucuronide	8.13	1.676-27.874	0.829	0.514-1.203	0.0391	0.039	3.293811345
2-Methylbutyroylcarnitine	6.221	2.587-648.963	0.001	0.001-0.552	0.0391	0.039	12.60293079
Tsugaric acid A	4.835	1.121-19.923	1.52	0.121-3.346	0.0391	0.039	1.669444566
7,8-Dihydrovomifoliol 9-[rhamnosyl-(1->6)-glucoside]	4.575	3.433-11.304	0.694	0.299-1.153	0.0078	0.0078	2.720764175

Guanine	4.534	1.643-10.04	1.66	1.054-3.149	0.0156	0.016	1.44960115
Isovalerylglutamic acid	4.431	1.973-13.975	0.958	0.665-1.291	0.0156	0.016	2.209534766
6,7-Dihydro-2,5-dimethyl-5H-cyclopentapyrazine	4.324	0.265-21.383	0.003	0.001-0.028	0.0234	0.023	10.49318831
N-Acetyldopamine	3.648	2.63-6.291	0.294	0.165-0.864	0.0234	0.023	3.633217669
N-Succinyl-L,L-2,6-diaminopimelate	2.982	0.96-172.502	0.026	0.001-0.142	0.0078	0.0078	6.841624824
Benzaldehyde	2.823	1.951-11.4	0.878	0.79-2.447	0.0234	0.023	1.684936284
2-Methyl-3-(2-methylpropyl)pyrazine	2.545	0.824-21.417	0.483	0.425-0.613	0.0391	0.039	2.397570562
7,8-Dihydro-3-methylpyrrolo[1,2-a]pyrimidin-2(6H)-one	2.369	2.081-2.922	1.052	0.906-1.336	0.0078	0.0078	1.171143494
Curcumin III	1.514	0.197-3.179	32.216	19.999-45.487	0.0391	0.039	-4.411340267
Ovalicin	1.22	0.38-35.102	0.001	0.001-0.002	0.0078	0.0078	10.25266543
N-Methylcalystegine B2	1.22	0.986-2.425	0.884	0.611-1.483	0.0391	0.039	0.464762873
Phaeophorbide b	1.006	0.069-3.221	0.013	0.008-0.036	0.0078	0.0078	6.273974872
(3R)-3,4-Dihydroxy-3-(hydroxymethyl)butanenitrile 4-glucoside	0.919	0.428-2.681	318.116	2.937-541.717	0.0391	0.039	-8.435272359
Equol	0.882	0.537-129.151	0.036	0.001-0.832	0.0391	0.039	4.614709844
(2S,2'S)-Pyrosaccharopine	0.871	0.444-1.603	0.259	0.144-0.315	0.0391	0.039	1.749720621
2,3-Dihydroxycarbamazepine	0.863	0.517-3.923	0.12	0.001-0.463	0.0156	0.016	2.846326154
Tryptophyl-Tryptophan	0.662	0.105-5.116	0.001	0.001-0.009	0.0078	0.0078	9.370687407
2-Fucosyllactose	0.631	0.051-3.452	6.972	5.542-11.063	0.0156	0.016	-3.465860659
trans-Caffeic acid [apiosyl-(1->6)-glucosyl] ester	0.501	0.001-4.159	16.946	4.753-43.119	0.0391	0.039	-5.079990361
5,7-dihydroxy-2-(4-hydroxy-3,5-dimethoxyphenyl)-4H-chromen-4-one	0.179	0.096-4.622	0.01	0.006-0.04	0.0391	0.039	4.161887682
ent-Epicatechin-(4alpha->6)-ent-epicatechin	0.152	0.001-0.505	3.36	1.362-3.898	0.0234	0.023	-4.466318004
Edulisin IV	0.125	0.031-0.542	5.265	0.852-15.559	0.0156	0.016	-5.396433531
2-(3,4-dihydroxyphenyl)-5-hydroxy-3,6,7-trimethoxy-4H-chromen-4-one	0.029	0.008-0.416	0.01	0.002-0.023	0.0391	0.039	1.5360529
1-O-Sinapoylglucose	0.001	0.001-0.016	7.01	0.34-31.211	0.0156	0.016	-12.77519873
PS(22:0/18:1(9Z))	0.001	0.001-0.169	0.312	0.011-1.725	0.0391	0.039	-8.285402219
Propionic acid	15.187	10.726-16.11	5.607	3.763-8.174	0.0391	0.039	-0.951520141
Cyclohexanecarboxylic acid	14.399	1.114-53.022	0	0-0.182	0.0078	0.0078	1.00E+05
3b-Hydroxy-5-cholenoic acid	11.478	6.468-32.216	3.11	2.227-6.772	0.0156	0.016	2.534152832
Deoxycholic acid	10.63	6.982-11.443	1.165	0.998-2.648	0.0078	0.0078	4.516556495
3-Sulfinoalanine	9.916	2.191-19.071	0.752	0-1.997	0.0078	0.0078	0.951820993

Capric acid	9.041	6.7-24.768	116.286	13.589-203.573	0.0391	0.039	-0.084275076
Gentisic acid	8.004	5.225-14.067	0.851	0.475-1.373	0.0234	0.023	-0.739692968
Terephthalic acid	6.004	5.34-6.124	5.189	4.847-5.462	0.0391	0.039	2.68291607
Phenylacetic acid	3.792	1.37-6.597	0.925	0.826-1.306	0.0078	0.0078	-0.146138093
3,4-Dihydroxybenzaldehyde	3.457	2.735-4.84	0.241	0.035-0.352	0.0156	0.016	0.044828304
Isohomovanillic acid	3.311	0.589-5.554	0.219	0.104-0.402	0.0156	0.016	0.213849896
D-Mannose	2.916	2.209-4.657	5.829	4.173-11.812	0.0156	0.016	0.098004255
N-Acetylleucine	2.686	1.286-6.985	0.637	0.361-1.281	0.0156	0.016	-0.51963416
N-Acetyl-L-phenylalanine	2.295	0.791-9.156	0.613	0.212-1.956	0.0391	0.039	0.891724629
4-Hydroxybenzaldehyde	2.141	1.643-9.279	1.378	1.274-1.729	0.0234	0.023	0.592848368
Byssochlamic acid	1.982	0.3-4.896	0.103	0.044-0.192	0.0234	0.023	-2.296539943
Maltitol	1.822	0.387-14.335	52.167	15.169-76.886	0.0781	0.078	-0.771993556
Azelaic acid	1.675	0.677-2.401	3.987	3.137-5.057	0.0391	0.039	0.229365848
Pyridoxine	1.569	0.497-3.819	3.539	1.071-16.611	0.0391	0.039	0.376215913
(1R,2S,3R)-2-Acetyl-4(5)-(1,2,3,4-tetrahydroxybutyl)imidazole	1.536	0.554-4.016	0.265	0.071-0.83	0.0781	0.078	0.348290591
(10E,12Z)-(9S)-9-Hydroperoxyoctadeca-10,12-dienoic acid	1.488	1.307-1.714	2.031	1.865-2.79	0.0234	0.023	-0.912310415
Leukotriene F4	1.417	0.063-3.199	0.004	0-0.086	0.0234	0.023	2.244330967
Indole-3-carboxylic acid	1.161	0.798-2.199	0.115	0.075-0.288	0.0156	0.016	-1.342907065
Glycolic acid	1.12	0.84-2.162	0.894	0.63-0.997	0.0391	0.039	2.129347725
3-Hydroxycapric acid	1.054	0.974-1.293	2.961	2.667-3.491	0.0156	0.016	-0.392326041
Malic acid	0.98	0.757-1.408	9.226	3.791-14.964	0.0156	0.016	1.530472743
Pentadecanoic acid	0.884	0.723-1.28	0.721	0.691-0.768	0.0234	0.023	-0.406779583
xi-2,3-Dihydro-2-oxo-1H-indole-3-acetic acid	0.864	0.638-5.938	0.101	0.037-0.294	0.0078	0.0078	-1.305710353
Deoxyinosine	0.783	0.46-1.768	1.615	0.695-7.307	0.0391	0.039	-0.155041923
3-Hydroxysebacic acid	0.427	0.171-0.652	1.401	1.381-1.629	0.0078	0.0078	0.893696951
Butyrylcarnitine	0.18	0.121-0.385	0.581	0.342-1.358	0.0391	0.039	-0.757269691
Melezitose	0.154	0.089-1.009	6.393	1.477-6.525	0.0078	0.0078	-0.024886588
Jaceidin	0.152	0.004-7.068	14.728	12.187-20.718	0.0156	0.016	1.536545372
D-Maltose	0.112	0.063-0.472	1.586	0.627-1.882	0.0156	0.016	0.133564365
Ginkgolide C	0.082	0.03-0.286	0.491	0.143-0.787	0.0391	0.039	0.74737736
Sucralose	0.003	0.002-1.405	280.387	169.795-366.218	0.0156	0.016	9.105908509
Monomethyl glutaric acid	0	0-0.257	0.716	0.057-1.119	0.0391	0.039	-0.501061243

Pre_KD, samples collected before ketogenic diet initiation; Post_KD, samples collected after one week of ketogenic diet therapy.

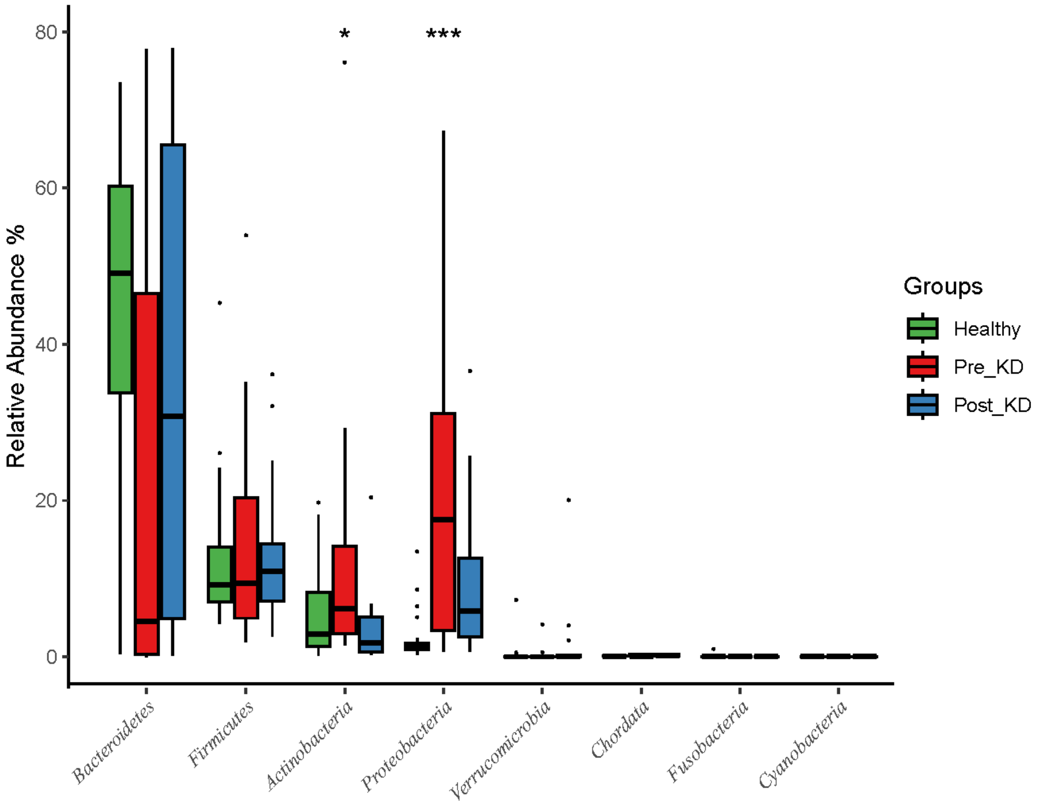

FIGURE S1 Comparison at phylum level. Pre_KD, samples collected before ketogenic diet initiation; Post_KD, samples collected after one week of ketogenic diet therapy.
